# Early adverse physiological event detection using commercial wearables: challenges and opportunities

**DOI:** 10.1038/s41746-024-01129-1

**Published:** 2024-05-23

**Authors:** Jesse Phipps, Bryant Passage, Kaan Sel, Jonathan Martinez, Milad Saadat, Teddy Koker, Natalie Damaso, Shakti Davis, Jeffrey Palmer, Kajal Claypool, Christopher Kiley, Roderic I. Pettigrew, Roozbeh Jafari

**Affiliations:** 1 Department of Computer Science and Engineering, Texas A&M University, College Station, TX USA; 2 Laboratory for Information and Decision Systems, Massachusetts Institute of Technology, Cambridge, MA USA; 3 Department of Electrical and Computer Engineering, Texas A&M University, College Station, TX USA; 4 Lincoln Laboratory, Massachusetts Institute of Technology, Lexington, MA USA; 5 Defense Threat Reduction Agency, Fort Belvoir, VA USA; 6 School of Engineering Medicine, Texas A&M University, Houston, TX USA

**Keywords:** Biomedical engineering, Health services, Infectious diseases

## Abstract

Data from commercial off-the-shelf (COTS) wearables leveraged with machine learning algorithms provide an unprecedented potential for the early detection of adverse physiological events. However, several challenges inhibit this potential, including (1) heterogeneity among and within participants that make scaling detection algorithms to a general population less precise, (2) confounders that lead to incorrect assumptions regarding a participant’s healthy state, (3) noise in the data at the sensor level that limits the sensitivity of detection algorithms, and (4) imprecision in self-reported labels that misrepresent the true data values associated with a given physiological event. The goal of this study was two-fold: (1) to characterize the performance of such algorithms in the presence of these challenges and provide insights to researchers on limitations and opportunities, and (2) to subsequently devise algorithms to address each challenge and offer insights on future opportunities for advancement. Our proposed algorithms include techniques that build on determining suitable baselines for each participant to capture important physiological changes and label correction techniques as it pertains to participant-reported identifiers. Our work is validated on potentially one of the largest datasets available, obtained with 8000+ participants and 1.3+ million hours of wearable data captured from Oura smart rings. Leveraging this extensive dataset, we achieve pre-symptomatic detection of COVID-19 with a performance receiver operator characteristic (ROC) area under the curve (AUC) of 0.725 without correction techniques, 0.739 with baseline correction, 0.740 with baseline correction and label correction on the training set, and 0.777 with baseline correction and label correction on both the training and the test set. Using the same respective paradigms, we achieve ROC AUCs of 0.919, 0.938, 0.943 and 0.994 for the detection of self-reported fever, and 0.574, 0.611, 0.601, and 0.635 for detection of self-reported shortness of breath. These techniques offer improvements across almost all metrics and events, including PR AUC, sensitivity at 75% specificity, and precision at 75% recall. The ring allows continuous monitoring for detection of event onset, and we further demonstrate an improvement in the early detection of COVID-19 from an average of 3.5 days to an average of 4.1 days before a reported positive test result.

## Introduction

Commercial off-the-shelf (COTS) wearables have gained significant popularity over the years^1,2^, enabling continuous access to physiological parameters such as heart rate, heart rate variability, skin temperature, and respiration rate, along with contextual information such as physical activity and sleep cycles^3–6^. There is an opportunity to leverage the extensive data acquired from COTS devices as an early detection mechanism for many physiological events, in particular responses to COVID-19, to mitigate future outbreaks and improve population health.

Recent studies^7–9^ have pursued detection of COVID-19 using COTS wearable devices, including Oura smart rings. However, despite the ability of wearables to capture continuous and real-time physiological information^10^, datasets obtained by these devices often suffer from a number of challenges when used for the early detection of infections: 1) inter- and intra-subject variability of physiological responses to the infection, such as differences in resting heart rate (HR) between an active and sedentary lifestyle^11,12^, 2) undesired confounders such as caffeine/alcohol intake or physical exertion that can lead to physiological responses similar to those caused by infection^13,14^, 3) sensor-level noise and artifacts such as noisy HR signals from motion or incorrect data from misplacement of the wearable^15,16^, and 4) less than ideal user-compliance for self-reported surveys^17–19^ leading to occasionally misleading labels for data-driven modeling approaches (see Supplementary Note 1 and Supplementary Figs. 1 and 2). To address the heterogeneity challenge, studies have proposed establishing a baseline window of historical data to represent healthy states and standardize physiological changes across participants^8^. However, with lack of contextual information, undesired confounders may corrupt baseline data^13^, misrepresenting the onset of infection. Furthermore, sensor-level noise may similarly affect baseline windows causing this error to propagate into detection performance^20^. Lastly, data-driven analytic solutions that map wearable data to physiological events require precise labeling that, for large-scale studies, are typically acquired through participant self-reporting^21^. However, less than ideal compliance is susceptible to inaccuracy due to delayed, incorrect, and missed reports. These incorrect event labels hinder detection performance by causing the model to fit to an incorrect representation of the reported physiological event.

In this study, we establish a set of robust data correction techniques that mitigate the impact of undesired confounders, sensor-level noise, and imprecise user-reported labels to further improve the early detection of infection using COTS wearables. The imprecision in user-reported labels could be caused by various factors including human errors, failing to report a label, reporting a label with a delay of one or more days, or not capturing correct labels (e.g., measuring body temperature prior to reaching a fever state of > 100.4℉ and hence reporting merely elevated body temperature label in lieu of user-reported fever label). We introduce a baseline correction algorithm applied as a preprocessing technique to obtain an improved estimate of a participant’s healthy state, resulting in better representations of physiological changes exhibited by that participant. We also introduce a label correction algorithm capable of correcting mis-reported symptom and/or infection labels that degrade detection performance. We leverage data from 8000+ participants and provide improved early detection of COVID-19 with a range of 7 days up to the night prior to a positive PCR test to further reduce the risk of outbreak in large populations. Our findings demonstrate an improved ROC AUC of 0.740 (sensitivity 62% at 75% specificity) and PR AUC of 0.636 (precision 43% at 75% recall) with baseline correction and label correction applied to the training set, and ROC AUC of 0.777 (sensitivity 66% at 75% specificity) and PR AUC of 0.688 (precision 42% at 75% recall) with baseline and label correction applied to both the training and testing sets. It is important to note that when label correction is applied to both testing and training, the visualization of the raw data (e.g., elevated temperature in presence of fever) clearly confirm that the labels were not correctly reported by the participants in a few instances (Fig. 5). Furthermore, we improve identification of infection on average 4.1 days prior to the COVID-19 positive test using COTS wearables, revealing their potential to be used as an early warning mechanism. We also provide analysis on the potential of these techniques for symptoms of COVID-19 as well, showing improvements in fever and shortness of breath detection. A principal contribution of this paper is to characterize various challenges as it pertains to COTS wearable data and offer insights to the community on expected outcomes and potential opportunities.

## Results

### Study design and overview

At the enrollment phase of data collection, basic demographic information and medical history for each participant was recorded. In addition, a daily survey that includes a series of questions to prompt each participant to report the onset of any infection, symptoms, medications, vaccinations, or COVID-19 testing results was distributed. PCR tests were administered frequently and not triggered by symptoms (see Supplementary Fig. 3), with a median time between tests of 6 days. Responses collected from the daily surveys were used as labels for training and testing and are not included in the feature set used for prediction. After data collection, our study consists of 8165 participants, with 6462 males and 1703 females aged 18–74 years. Of the participants in our cohort, 358 tested positive for COVID-19, 183 of whom were symptomatic. Furthermore, 1637 participants reported a negative result for a COVID-19 test.

**Fig. 1:**
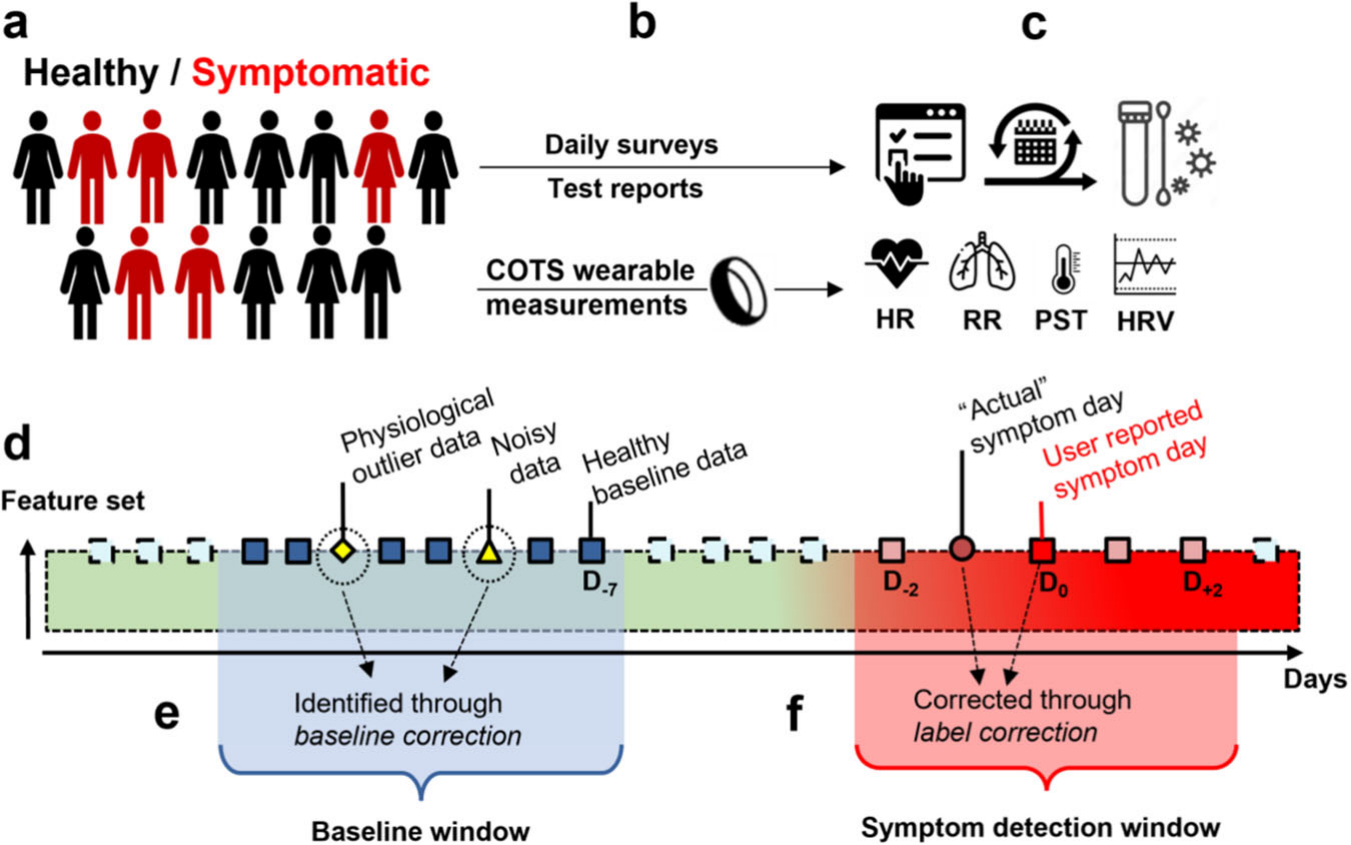
Overview of the study experiment and design. **a** Participants recruited with demographic information. **b** Participants submitted daily surveys of any symptoms or test reports and smartring data extracted from participants for (**c**), the physiological information captured. This information includes heart rate (HR), respiration rate (RR), peripheral skin temperature (PST), and heart rate variability (HRV). The information from the submitted surveys captured any reported symptoms, COVID-tests, and additional measurements related to device measurements. **d** Feature values are extracted from recorded physiological measurements and baselines and evaluation windows are calculated. Within baseline window calculation, (**e**) remove anomalous nights for best representative healthy baseline. **f** Correction of labels within each evaluation window. After correction, the machine learning model is trained with the corrected baseline and label data.

The wearable used for continuous physiological data acquisition is the Oura Ring Gen2 by Oura Ltd. (Oulu, Finland). The Oura Ring provides heart rate (HR), interbeat interval (IBI), root mean square of successive differences (RMSSD, i.e., a measure of HRV), peripheral skin temperature (PST), coarse-grained activity, and sleep reports (Fig. 1b, c). Oura obtains these physiological parameters only during periods of sleep with the exception of peripheral skin temperature which is continuously collected throughout the day and night. Moreover, we derive additional features from the collected IBI stream, such as respiration rate^22^ (RR) (see Methods).

We utilize machine learning (e.g., XGBoost^23^) models for the prediction of physiological events. Features are extracted from the raw data captured by COTS wearables and then corrected using the baseline correction (BC) algorithm (Fig. 1d, e). Simultaneously, label correction (LC) is applied to the labels provided by the participants (Fig. 1f). For the purposes of this study, we evaluate four framework configurations: 1) conventional machine learning (ML) framework with no preprocessing correction applied, 2) baseline correction applied prior to training, 3) baseline correction and label correction applied to the training set, and 4) baseline correction and label correction applied separately to both the training and testing set (Table 1) to demonstrate the improvements and limitations of our techniques. We further emphasize that configuration 4 is not indicative of real-world performance, but rather an indication of performance when labels within the test set are statistically similar. The effects of this label correction on the test set are analyzed in the discussion section of this paper.

**Table 1:** Data correction frameworks used in this study and their descriptions

Type	Description
Conventional ML Framework	Standard machine learning approach to detect COVID-19 prior to the onset of symptoms. The classification algorithm used is the XGBoost Classifier.
Baseline Correction (BC)	Correction of baseline windows prior to training and testing of the ML model used in the conventional framework.
BC and LC on Training	Baseline correction applied to the data and the correction of user-reported symptom/diagnosis labels in the training set used to train the ML model in the conventional framework.
BC and LC on Training and Testing	Baseline correction applied to the data and the correction of user-reported symptom/diagnosis labels in the training set and test set used to train/evaluate the ML model in the conventional framework.

### Data inclusion

For analyses, we only include participants’ data who had at least two days of valid data within the seven days leading to a positive COVID-19 test and valid data the night before the participant reported a symptom. This is enforced to train and test the models on data that are close to the reported symptom/diagnosis label without gaps in evaluation windows. Negative, or healthy, windows are derived from periods before the first positive event for each participant, with a 2-week offset. These negative windows are from periods without any reported symptoms or positive COVID-19 results and are the same length as positive windows to ensure the proper calculation of metrics. We use these windows to evaluate the efficacy of our models and techniques which allow for prediction of COVID-19 prior to diagnosis and detection of symptoms related to COVID-19 (see Methods). No participant data were ever present in both training and testing simultaneously and no participant was ever selected for multiple testing folds. Out of the 358 COVID-19 positive cases, 141 positive cases follow the data inclusion criteria. Furthermore, user symptom reports are also evaluated, with 281 users reporting fever and 184 reporting shortness of breath. Of these reports, a subset of 140 and 86 users are selected for symptom detection analysis due to the same inclusion criteria. In total, 357 negative and 141 positive evaluation windows are included for COVID-19, 310 negative and 86 positive evaluation windows for shortness of breath, 442 negative and 96 positive evaluation windows for elevated temperature, and 236 negative and 44 positive evaluation windows for fever. All negative and positive evaluation windows were selected from participants that had at least one (1) positive evaluation window. This was to enforce that there existed both positive and negative evaluation windows for each participant, ensuring an evaluation of event detection at both the participant and population levels. It should be noted that because of this, positivity rates in the selected participants’ data may not match those found in other works such as Natarajan et al. ^9^. The supplementary analysis shown in Supplementary Note 2 and Supplementary Table 1 explores the false-positive rates (FPR) obtained when expanding the COVID-19 cohort to participants without a positive evaluation window.

### Early detection of COVID-19

For the early detection of COVID-19, the conventional framework achieves a ROC AUC of 0.725 and a PR AUC of 0.619, providing a sensitivity of 60% at specificity of 75%, and precision of 40% at recall of 75% when noise mitigation techniques are not applied. With only baseline correction applied, the performance improves to a ROC AUC of 0.739 (1.4% absolute improvement), and a PR AUC of 0.638 (1.9% absolute improvement), providing a sensitivity of 61% at specificity of 75%, and precision of 43% at recall of 75%, an improvement of 1% and 3% for sensitivity and precision respectively. With baseline correction applied and label correction applied to the training set, we achieve an increased ROC AUC of 0.740 (3.5% absolute improvement), and a PR AUC of 0.636 (1.7% absolute improvement), providing a sensitivity of 62% at specificity of 75%, and precision of 43% at recall of 75%, an improvement of 8% and 8% for sensitivity and precision respectively. Furthermore, when baseline correction and label correction are applied to the training and testing set, the performance increases to ROC AUC of 0.777 (5.2% absolute improvement), and a PR AUC of 0.688 (6.9% absolute improvement), providing a sensitivity of 66% at specificity of 75%, and precision of 42% at recall of 75%, an improvement of 6% for sensitivity and 2% for precision (Table 2). Additional analysis on symptomatic vs. asymptomatic COVID-19 cases can be viewed in Supplementary Note 3 and Supplementary Table 2.

**Table 2:** Performance of each symptom and COVID test diagnosis with data correction techniques applied to the training set

	Metric	Symptom / Diagnosis			
		COVID-19	Fever	Elevated Temperature	Shortness of Breath
Conventional Framework	ROC AUC	0.725 (0.678, 0.772)	0.919 (0.886, 0.952)	0.832 (0.799, 0.864)	0.574 (0.497, 0.652)
	PR AUC	0.619 (0.577, 0.660)	0.877 (0.852, 0.902)	0.693 (0.641, 0.746)	0.294 (0.252, 0.337)
	Sensitivity*	0.598 (0.556, 0.641)	0.883 (0.862, 0.904)	0.775 (0.747, 0.803)	0.313 (0.221, 0.404)
	Precision*	0.399 (0.345, 0.452)	0.809 (0.616, 1.002)	0.469 (0.382, 0.556)	0.280 (0.247, 0.313)
Baseline Correction	ROC AUC	0.739 (0.682, 0.797)	0.938 (0.892, 0.985)	0.854 (0.803, 0.905)	0.611 (0.521, 0.702)
	PR AUC	0.638 (0.592, 0.683)	0.889 (0.851, 0.927)	0.722 (0.697, 0.748)	0.334 (0.225, 0.444)
	Sensitivity*	0.613 (0.559, 0.666)	0.920 (0.887, 0.952)	0.806 (0.781, 0.831)	0.420 (0.319, 0.521)
	Precision*	0.429 (0.384, 0.473)	0.862 (0.734, 0.989)	0.515 (0.446, 0.584)	0.282 (0.228, 0.335)
BC + LC on Training	ROC AUC	0.740 (0.680, 0.799)	0.943 (0.901, 0.985)	0.864 (0.804, 0.924)	0.601 (0.514, 0.688)
	PR AUC	0.636 (0.586, 0.687)	0.879 (0.826, 0.932)	0.738 (0.712, 0.765)	0.314 (0.248, 0.379)
	Sensitivity*	0.618 (0.591, 0.645)	0.933 (0.904, 0.962)	0.807 (0.785, 0.829)	0.400 (0.310, 0.490)
	Precision*	0.433 (0.385, 0.481)	0.810 (0.715, 0.906)	0.516 (0.471, 0.561)	0.285 (0.250, 0.320)
BC + LC on Training + Testing	ROC AUC	0.777 (0.710, 0.844)	0.994 (0.951, 1.038)	0.940 (0.872, 1.009)	0.635 (0.530, 0.741)
	PR AUC	0.688 (0.629, 0.748)	0.970 (0.891, 1.049)	0.877 (0.770, 0.984)	0.327 (0.253, 0.400)
	Sensitivity*	0.655 (0.607, 0.703)	1.000 (1.000, 1.000)	0.932 (0.874, 0.990)	0.426 (0.296, 0.557)
	Precision*	0.419 (0.368, 0.470)	0.963 (0.898, 1.028)	0.815 (0.689, 0.942)	0.258 (0.202, 0.315)

^*^Sensitivity and precision assessed at 75% specificity and 75% recall, respectively.

() Paratheses indicate the upper and lower bounds of the calculated 95% confidence interval (μ ± 1.96 × σ).

Additionally, on average, the conventional model predicts physiological events associated with COVID-19 3.5 days before the COVID-positive test. With baseline and label correction applied to the data, the model predicts positive cases on average 4.1 days before a COVID-positive test, supporting how noise can also impact the temporal prediction probability relative to a positive test. In the case of the full data correction framework, there was a shift in the prediction of more cases earlier within the 7-day period than with the conventional model, with 55 positive cases being predicted at day -6 and -5 from 26 detected positive cases previously. This shift in earlier detection demonstrates the framework’s ability to better distinguish marginal class differences between positive and negative cases of COVID-19 compared to models fit to noisier distributions (see Supplementary Note 4).

### Fever and shortness of breath detection

In addition to COVID-19 prediction, we analyze two common symptoms associated with COVID-19 – fever and shortness of breath (SOB) – and show the conventional framework performance of the classification model and the improvements brought by the proposed data correction techniques. Furthermore, each participant that reported fever included a measured oral temperature of either above or below 100.4 ℉. We categorize reported fevers with a temperature above this value as fever as per CDC guidelines^24^. The reported fevers with temperature ranges in 99.1–100.3 ℉ are categorized as elevated temperature cases. Fever and SOB exhibit diverse physiological responses and demonstrate varying difficulty in detection when using information captured by COTS wearables^24,25^. Thus, it is beneficial to demonstrate the efficacy of the proposed correction techniques when attempting to detect fever and SOB and how model performance can improve through a corrected dataset. In contrast to early detection of COVID-19, we detect the onset of fever and SOB rather than predict it. Therefore, evaluation windows for these symptoms are centered around the user-reported label rather than before the label (see Methods).

**Fig. 2:**
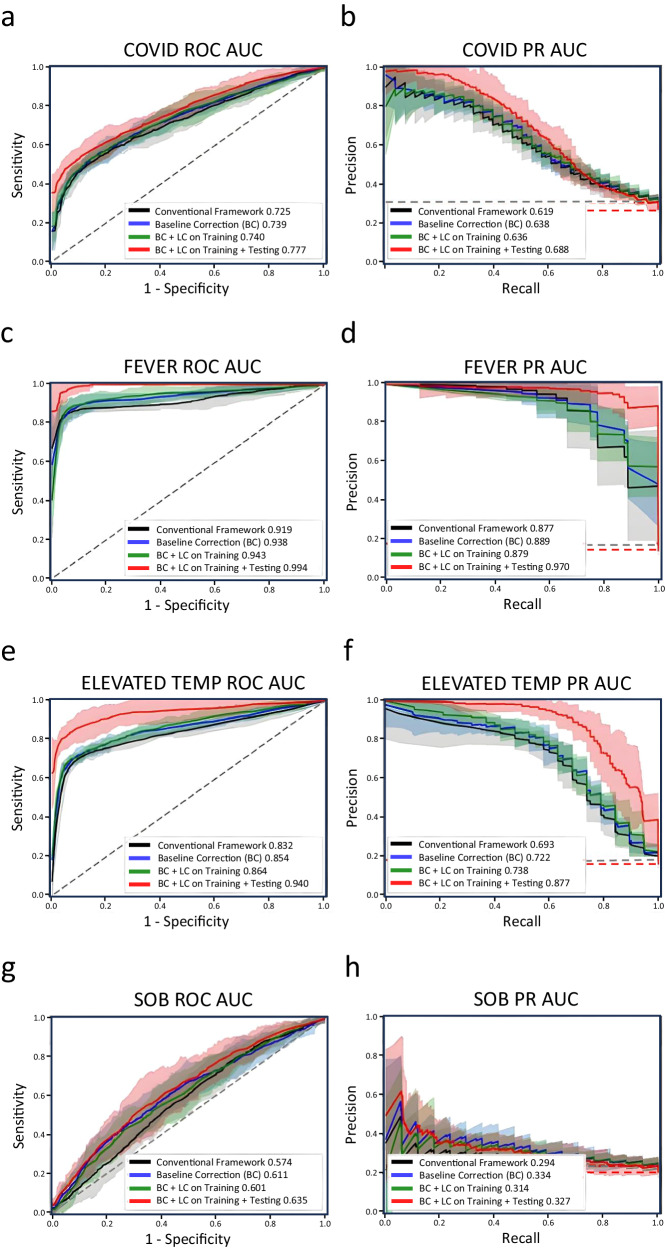
ROC and PR curves on model performance before and after data correction. ROC and PR curves for (**a**, **b**), COVID-19, (**c**, **d**), fever, (**e**, **f**), elevated temperature, and (**g**, **h**), shortness of breath across conventional framework, baseline correction, label correction, and full framework performance. The black curve shows the performance of the conventional framework with no correction algorithms applied. The blue curve shows the performance of the conventional framework when baseline correction is applied to the dataset. The green curve shows the performance with baseline correction applied to the dataset and label correction is applied on the training set. The red curve shows the performance when baseline correction and label correction is applied on both the training set and testing set. The shaded region of each curve represents the 95% confidence interval across 10 unique 5-fold splits (*n* = 10) (*μ ±* 1.96 × *σ*). The gray and red dashed lines in all three PR curves indicate the no skill ratio. The gray no skill line indicates the ratio when no labels are corrected in the test set. The red no skill line indicates the ratio when labels are corrected in the test set. The decrease in no skill between the original test set and corrected test set is due to the decrease of positive labels from features that are non-representative of symptoms or diagnosis.

For the detection of fever, the conventional framework achieves a ROC AUC of 0.919 and a PR AUC of 0.877, providing a sensitivity of 88% at a specificity of 75%, and precision of 81% at a recall of 75% without any noise mitigation applied (Fig. 2). With only baseline correction applied, we achieve an increased ROC AUC of 0.938 (1.9% absolute improvement), and a PR AUC of 0.889 (1.2% absolute improvement). It achieves a sensitivity of 92% at a specificity of 75%, and precision of 86% at a recall of 75%, an increase of 4% in specificity and 5% in precision. With baseline correction applied and label correction applied to the training set, we achieve an increased ROC AUC of 0.943 (2.4% absolute improvement), and a PR AUC of 0.879 (0.2% absolute improvement), providing a sensitivity of 93% at a specificity of 75%, and precision of 81% at a recall of 75%, an improvement of 5% and 0.1% for sensitivity and precision respectively. Furthermore, when baseline correction and label correction are applied to the training and testing set, the performance increases to ROC AUC of 0.994 (7.5% absolute improvement), and a PR AUC of 0.970 (8.7% absolute improvement), providing a sensitivity of 100% at a specificity of 75%, and precision of 96% at a recall of 75%, an improvement of 12% for sensitivity and 15% for precision (Table 2). Elevated temperature cases are also analyzed, and corresponding metrics can be viewed in Table 2. In addition to the analysis on fever and elevated temperature detection, we show the difficulty in detecting slight elevations in temperature over fever in Supplementary Note 5 and Supplementary Fig. 4 which shows the Z-score values of the most contributing features to the model for both fever and elevated temperature cases.

For SOB detection, the conventional framework achieves a lower ROC AUC of 0.574 and a PR AUC of 0.294, providing a sensitivity of 31% at a specificity of 75%, and precision of 28% at a recall of 75%. With baseline correction applied, performance increases ROC AUC to 0.611 (3.7% absolute improvement), and PR AUC of 0.334 (4% absolute improvement). It then achieves a sensitivity of 42% at a specificity of 75%, and precision of 28% at a recall of 75%, an increase of 11% in sensitivity. With baseline correction applied to the entire dataset and label correction applied onto the training data, we improve the detection performance with an increased ROC AUC of 0.601 (2.7% absolute improvement), and a PR AUC of 0.314 (2% absolute improvement), providing a sensitivity of 40% at a specificity of 75%, and precision of 29% at a recall of 75%, an improvement of 9% sensitivity and 0.5% precision. Lastly, when baseline correction and label correction are applied to the training and testing set, the performance increases to a ROC AUC of 0.635 (6.1% absolute improvement), and a PR AUC of 0.327 (3.3% absolute improvement), providing a sensitivity of 43% at a specificity of 75%, and precision of 26% at a recall of 75%, an improvement of 12% for sensitivity and a decrease of 2% for precision (Table 2). It should be noted that when correcting the labels in the test set, some positive labels are dropped, decreasing the no skill ratio in the precision-recall metrics shown.

### Visualization of label correction effects

**Fig. 3:**
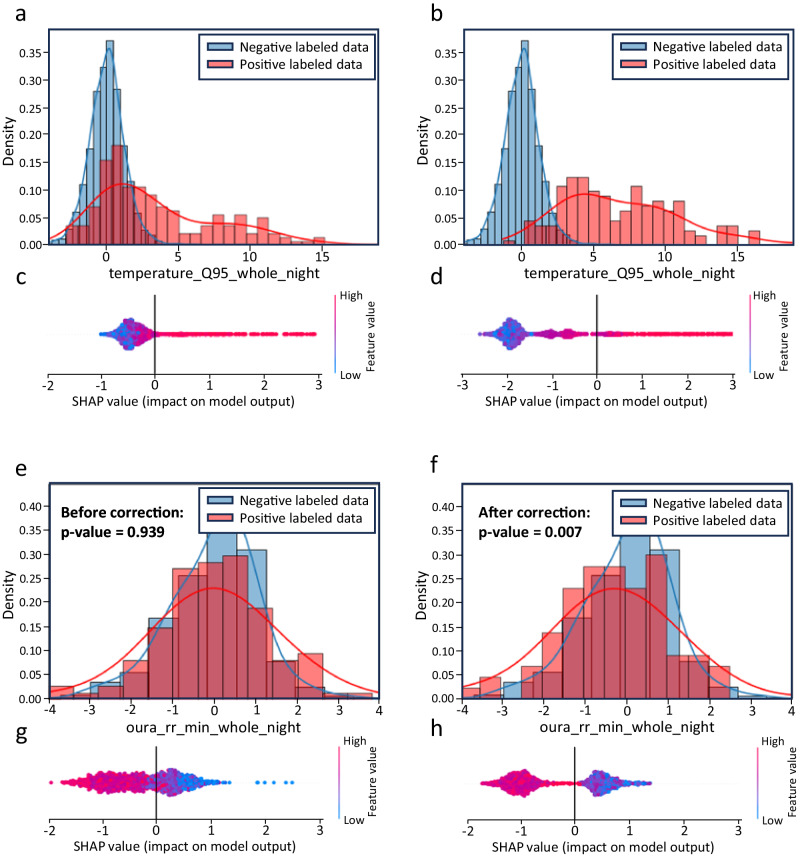
High-importance feature distributions between negative and positive samples in the training set and their corresponding SHAP values before and after label correction for fever and SOB. **a** Distributions of normalized 0.95 quantile temperature values for negative (blue) and positive (red) samples in the training set before label correction and (**b**), distributions of normalized 0.95 quantile temperature values in the training set after label correction. **c** SHAP values for temperature values in the training set before label correction and (**d**), SHAP values after label correction. **e** Distributions of normalized minimum respiration rate in the training set before label correction and (**f**), after label correction on the training set. **g** SHAP values for minimum respiration rate values in the training set before label correction and (**h**), after label correction on the training set. *P*-values were obtained for respiration rate data by conducting a two-sided t-test between positive (*n* = 122 before correction, *n* = 86 after correction) and negative labeled (n = 1481 before correction, *n* = 1481 after correction) samples.

To better understand the effect label correction has on training data, we perform feature distribution analyses and SHapley Additive exPlanations (SHAP) on the set before and after correction. Figure 3 demonstrates an example of how the correction process affects the top contributing feature (according to SHAP) of shortness of breath and fever: the normalized minimum respiration rate and normalized 0.95 quantile peripheral skin temperature, respectively, as shown in Fig. 3. The distributions for both cases show improved separation after label correction, which led to higher separability in their corresponding SHAP values after fitting a model. This demonstrates a consensus being reached by the algorithm as to what should and should not be considered a positive sample, as there is less “gray area” in SHAP values across samples.

### Early probabilistic prediction of adverse events

**Fig. 4:**
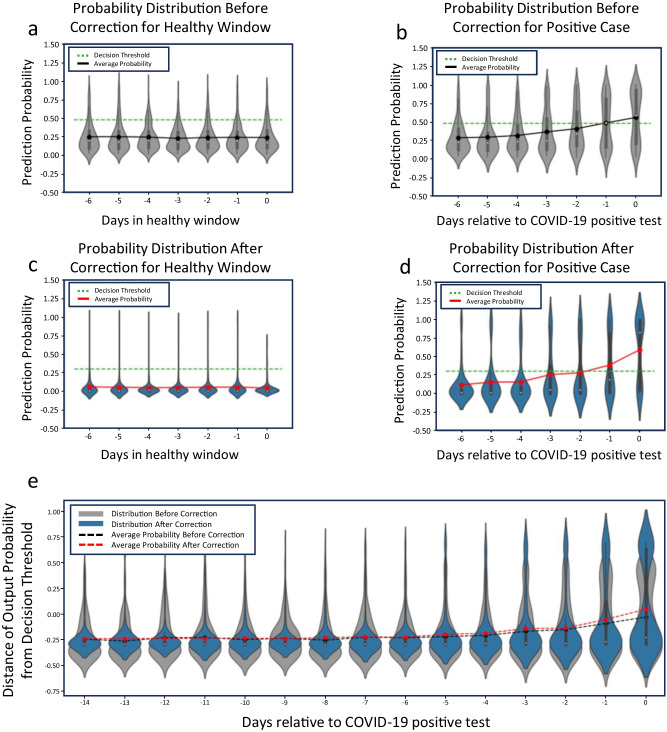
Model output prediction probabilities before and after data correction. Machine learning model prediction probabilities for (**a**), days within a healthy window without data correction, **b**, days leading up to COVID-19 positive test without data correction, (**c**), days within a healthy window with data correction and (**d**), days leading up to COVID-19 positive test with data correction. **c** shows more confident prediction probabilities from the model for days in a healthy window with concentrated predictions at lower probabilities around 0.07 compared to (**a**), which had an average prediction probability of around 0.3. **d** also supports higher prediction confidence closer to the positive test date compared to (**b**). **e** Shows the distance of output probability from the respective decision threshold of the models with and without data correction. **e** Supports strong negative predictions at early stages of COVID-positive tests for both models with an earlier increase in output probability for the model trained on corrected data, allowing for further COVID-positive predictions. The green dotted line shows the decision threshold at 75% recall for each model.

We analyze the confidence of the framework and how COVID-19 prediction probabilities change with respect to the data being corrected by our algorithms. We evaluate the data correction framework and conventional framework with the same classifier parameters and with a set recall of 75%, fixing each model with an equal number of predicted positive cases. As such, the decision threshold (threshold of the model’s output score at which cases are classified as positive or negative) reflects the set recall. For predictions made by the model in a negative evaluation window, the framework trained with corrected data outputs lower prediction probabilities, increasing confidence in decisions for negative cases (Fig. 4a, c). Model prediction probabilities on days close to a COVID-19 positive test show wider distributions in probabilities, however, there is a more substantial increase in average prediction probabilities found on the model trained with corrected data (Fig. 4d) over the model trained on the original data (Fig. 4b). Figure 4e further shows how the output probabilities from the model can affect prediction of infection on a rolling basis. We extend the number of days leading to a COVID-positive test up to 14 days prior to the test and scale the probabilities with the distance relative to the decision threshold of the model at 75% recall. During earlier stages of infection, the model output probability is similar for the framework with data correction. However, the model gains confidence earlier in the window, at approximately day -5, where output probability is higher compared to the model without data correction. The average probability continues to increase until the day before the COVID-positive test where the distance of the output probability from the decision threshold for the model trained on corrected data is highest. While this early probabilistic prediction shows potential for early mitigation of infectious diseases, lowering thresholds for earlier prediction increases the likelihood of false positives. Thus, these tradeoffs should be weighed by designers and stakeholders depending on the final application.

## Discussion

In 2020 alone, COVID-19 led to more than 2 million deaths worldwide^26,27^, increasing the urgency for infection identification and prevention in large cohorts. With over 30% of the US adult population utilizing some sort of wearable health device and over half using them daily^2^, COTS wearables provide a unique opportunity of mitigating health risks in the general population, especially in the role of detecting early onsets of infection. Although COTS wearables have a number of gaps and challenges when used with data-driven models, we propose data correction algorithms that address these challenges. To improve the ability of commercially available wearables to provide early detection of infections, we implement two preprocessing correction algorithms – baseline correction and label correction – that mitigate the contextual, sensor, and label noise associated with comprehensive COTS datasets. Through this case study, we found increased performance in data-driven models when both correction techniques were combined, demonstrating the potential for these correction techniques to be used as a preprocessing approach to machine learning models that leverage user self-reported labels and datasets that require baselining and standardization across different heterogeneous factors. Additionally, we provide analysis on the limitations of the techniques as well as the data, presenting insights into future opportunities to improve COTS wearable datasets.

The XGBoost classification algorithm was selected for this study due to its proven ability to classify tabular data and wide use in machine learning applications. The feature space consists of extracted statistical information including quantiles ranging from 0.05 to 0.95, mean, min, max, and standard deviation of the measurements recorded from the smart ring to allow for distributional information to be utilized by the model.

As stated previously, data from COTS wearables suffer from contextual and sensor-level noise. Our baseline correction addresses these specific challenges by correcting baselines through removal of nights that represent contradictory physiological information compared to other nights in the baseline window. This is achieved through pattern identification of physiological measurements capable of capturing statistical anomalies and trends between data observed in each night. This anomaly detection functionality can be deployed to seek further contextual information from users of COTS wearables (e.g., physical exertion, fatigue, stress) and gain insight to the user’s health state^28,29^. Furthermore, it may also be able to distinguish certain categories of contextual information from the physiological information captured by wearables, although this needs to be further studied and requires daily reported activities and lifestyles of participants for training models. While baseline correction generally improves detection performance, there also exist limitations resulting from 1) the constraints of the baseline window and 2) the correlation of the feature space to the noisy labels. Statistically, more samples in the baseline window mitigate the effect of outliers in the standardization of physiological information^30,31^. However, having a larger sample of nights required in baseline windows also extends the time a participant needs to continuously wear a device before any functionality is available. An analysis was performed on the optimal number of days needed in a participant’s baseline and it was determined that a 14-day baseline with a minimum of 7 days is sufficient for the standardization procedure (see Supplementary Note 6 and Supplementary Fig. 5). Furthermore, correcting the baseline does not address imperfections in label reports used for model training and classification. Correcting baselines may improve the associated feature values used for model prediction, although if the labels corresponding to those improved feature values are incorrect, model training may be negatively impacted. Although baseline correction itself can improve detection performance, applying label correction with baseline correction can significantly improve the overall performance.

**Fig. 5:**
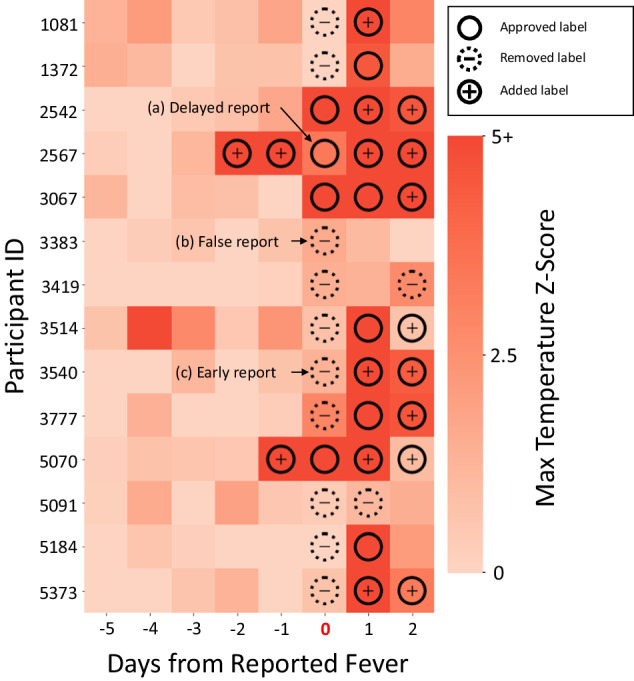
Fever labels before and after correction. Participant temperature data leading up to a fever report (day 0), with participant data separated on the y-axis and days from the report represented on the x-axis. Each square represents a single night of temperature data for the corresponding participant. The color indicates the maximum temperature Z-score, with approve, remove, and added fever labels overlayed. Approved labels are labels that were originally reported by the participant and agreed upon by the correction algorithm, removed labels are ones reported by the participant but not agreed upon by the correction algorithm, and added labels are fever labels that were added to the participant’s data by the correction algorithm. **a** An example of a delayed report, in which temperature onset occurred before the fever was reported. **b** An example of a false report, in which no meaningful indicators of fever could be found around the time of report. **c** An example of an early report, in which indicators of fever are delayed in the participant’s data.

Figure 5 demonstrates the need for label correction, where inaccurate labels can be caused by offsets in reporting time (Fig. 5a, c) or poorly representative/false reports (Fig. 5b). By leveraging the data within a window around a time of report, the information in a single dataset (either training or testing) can be used to reach a consensus as to what should and should not be considered a positive case. Our label correction method was shown to successfully improve performance across early COVID-19, fever, and SOB detection, both with and without correction of the test set. Figure 3 then demonstrates the benefits of our correction method in the training data, where physiologically related features are used to further distinguish between negative and positive cases. Supplementary Fig. 6 also demonstrates improved alignment of feature trajectories leading up to fever onset after applying our label correction method, particularly for temperature trajectories. Supplementary Fig. 7 depicts a correlation matrix of covariates, showing increased correlations after label correction is applied to the set. An outline of this analysis is provided in Supplementary Note 7. This allows us to remain confident in the algorithm’s ability to extract meaningful, physiologically relevant information from the training set and apply it to the task of label correction by leveraging the algorithm’s semi-supervised approach.

Experimentation on synthetically injected noise on the dataset was performed to measure the robustness of the label correction algorithm in response to varying levels of noise. It was found that labels in the corrected set demonstrated higher resistance to noise as compared to labels in the original dataset. This analysis can be found in Supplementary Fig. 8.

Even after extensive correction of the training set, the detection performance improvement across events is still limited. While this may partly be a limitation of the correction algorithms, the distribution of class features and labels between the corrected training sets and the original testing sets no longer accurately represent the same characteristics, resulting in inaccurate predictions in the test set. We demonstrate this after applying label correction to the test set as well as the training set, observing high detection performance across all events. Although modifying the labels in the test set is not necessarily indicative of performance in real-world applications, it presents meaningful insights into the limitations of performance due to label noise in the test set (Fig. 3). It should be noted that the label correction algorithm only utilizes information within the dataset it was correcting (either training or testing), and thus data leakage was avoided for both cases. Furthermore, rather than excluding evaluation windows from the test set that are determined to be falsely positive, these windows are given negative labels. This enforces the importance of reliability when correcting the test set, as poor corrections can lead to poor performance when evaluating on the new labels. Supplementary Fig. 9 shows which labels were converted for the test set of fever based on the top contributing feature for detection. Correction of the data prior to training can produce model prediction improvement; however, if the testing set labels excessively misrepresent the physiological features gathered by wearables, then model performance is limited to the accuracy of the labels in the test set. Our analysis on the performance after correction of the test set supports this observation as correction of the test set better represents the physiological responses learned by data-driven models. This presents an opportunity for future studies that leverage self-reported labels to focus on obtaining more accurate user-reported data and promote user-compliance towards surveys conducted during the study.

Additionally, we show the impact of these correction algorithms in improving the detection of symptomatic events, namely fever and SOB, demonstrating possible early detection of other infectious diseases such as pneumonia^32^, tuberculosis^33^, and influenza^34^.

We also investigate the discrepancy in performance between detection of fever and detection of SOB. Fever is a precisely defined symptom. The Centers for Disease Control and Prevention (CDC) defines it as an elevation in core body temperature above 100.4℉^24^, whereas diagnosis of SOB is often subjective^25^. This can result in more diverse representations of SOB in wearable datasets hindering the ability of data-driven models to detect the symptom. Future work on detection of SOB should build on recent work focusing on capturing vocal and physiological data during SOB while simultaneously categorizing its underlying cause^35,36^. While fever is precisely defined, it is not a trivial detection task. Wearables capture peripheral skin temperature (PST) rather than core body temperature (CBT). Furthermore, PST and CBT have been shown to be inversely correlated around periods of sleep^37^. Thus, absolute core body temperature cannot be determined from such measurements but is rather estimated.

Finally, we compare our methods to other off-the-shelf label correction algorithms to benchmark our data correction techniques, and our methods outperformed other off-the-shelf methods across ROC AUC and PR AUC for all symptoms and COVID-19 except for PR AUC of SOB. These results can be seen in Supplementary Table 3 with their corresponding hyperparameters in Supplementary Table 4.

A future opportunity for this work is the analysis of a symptom report-based model for COVID-19 early detection as seen in existing studies^38^, and its comparison with the use of COTS wearables for such tasks.

The results of this unique case study highlight the challenges, importance, and benefits of mitigating domain-specific noise in wearable health datasets. Our findings indicate improvements in data-driven analytics for the early detection of COVID-19 and two associated symptoms: fever and shortness of breath. Although the application of our baseline and label correction techniques were limited to this case study, the concept of utilizing these techniques has wider applicability beyond the early detection of COVID-19.

## Methods

### Data source

This study was approved by the following institutional review boards: Clinical Investigation Department Naval Medical Center, San Diego CIP #NMCSD.2020.0027; and Air Force Research Laboratory Wright-Patterson Air Force Base, Ohio #WR20200175H. All participants provided written informed consent.

Wearable sensor data was collected by an Oura Ring Gen2 by Oura Ltd. (Oulu, Finland). The Oura Ring collects skin temperature, HR, IBI, HRV RMSSD, activity intensity, and sleep information, however, HR, IBI, and HRV RMSSD are only provided during sleep while temperature and activity intensity are provided continuously over 24 h to account for motion artifacts.

### Data preprocessing

Data from the Oura Ring are segmented into nightly segments based on the Oura Ring’s detected sleep segment timestamps of each night. Processing and analyzing only the nocturnal physiological data collected by the wearable devices avoids possible motion artifacts that disrupt nightly physiological trends over time and allows for analysis of physiological information when the participant is at rest.

Interbeat interval (IBI) is defined as the time interval between successive heart beats. With IBI, we can further extract respiration rate (RR) through its frequency components^39,40^.

From each segment, we extract statistical features such as quantiles which quantify the overall distribution of the data and other features including mean, standard deviation, kurtosis and skewness which summarize the characteristics of the distribution.

### Baseline calculation

Once features have been extracted per night, the data needs to be standardized to remove inter-subject variability. For each segment data, we define a 14-day window with a 7-day gap in the past as the baseline for the data. The window size was chosen to be 14 days to allow for ample opportunity to capture a healthy baseline, while a 7-day gap was enforced to ensure that data within the baseline window do not experience symptom onset before the actual event that could cause changes to physiological data that could affect the baseline. To maximize data availability, the 14-day baseline should meet the following conditions to be considered valid: 1) there are no symptoms reported within the window and 2) there were at least 7 days of valid data within the window (see Supplementary Note 6 and Supplementary Fig. 5). Therefore, the window size can have anywhere between 7 and 14 days of nightly data. After extracting a valid baseline window for data segment *D*_*i*_, features *x*_*j*_ for *j* = 1,..,*m* – where *m* is the number of features extracted – will then be standardized by the mean *μ*_*j*_ and standard deviation *σ*_*j*_ of the baseline window using the following equation:

1 $${Z}_{j}=\frac{{x}_{j}-{\mu }_{j}}{{\sigma }_{j}}$$

The mean *μ*_*j*_ and standard deviation *σ*_*j*_ are calculated for each feature across all the segmented data within the baseline window. We use Z-scores from which personal baselines are removed, as input to train and test the classifier.

### Evaluation windows

To mitigate the effects of user-reported label noise when evaluating performance, data samples were windowed based on their temporal proximity to one another. For example, a single window may consist of 5 samples, each one being a single night of data, N_i_. Windows are denoted as either negative and positive, where positive windows are made up of nights on or around a reported event, and negatives have no reported events of any kind during any night in the window. For symptoms (e.g., SOB, fever), windows are centered around the reported event, with two-night buffers before and after the night it was reported on. If a window is centered around an event on night N_0_, the entire window would comprise nights N_-2_, N_-1_, N_0_, N_1_, and N_2_. If multiple nights were reported for a single event, for example on nights N_0_ and N_2_, then the two-night buffers are before N_0_ and after N_2_, resulting in a window of nights N_-2_, N_-1_, N_0_, N_1_, N_2_, N_3_, and N_4_. One positive window is considered for each participant, where data after the first positive window is excluded from the dataset. To ensure prediction methods do not bias results towards positive windows, each negative window in a user’s data has the same number of nights as the positive window for that user. Windowing for COVID-19 prediction uses the same principle, however, since the objective is now prediction, we comprise positive windows with 7 nights leading up to COVID-19 diagnosis (e.g., N_-6_, N_-5_, N_-4_, N_-3_, N_-2_, N_-1_, N_0_, with N_0_ as the night before the user reported a positive COVID-19 test). Defining COVID-19 evaluation windows in this way agrees with previous reports indicating up to one-week duration of symptomatic response after exposure^41^. Therefore, it is assumed that a physiological response occurs within the one week-window prior to each positive diagnosis. Negative COVID-19 windows are generated using the same procedure for negative fever and shortness of breath windows with the exception that negative COVID-19 windows are 7 nights long to avoid detection bias towards positive COVID-19 windows. It should be noted that N_0_ is the night before a symptom label or COVID-19 diagnosis label and therefore is still considered an early prediction night. Any *i* > 0 is considered detection. See Supplementary Fig. 10 for more details on evaluation windows.

### Baseline correction via physiological pattern identification

The aim of physiological pattern identification is to identify and discard abnormal nights from healthy baselines. Within a 14-day baseline, a user may exhibit various nightly physiological patterns that reflect the quality of sleep he/she may experience. Identifying the healthy nightly patterns and only utilizing those for the baseline can increase the accuracy of detection. For this task, we utilize a variational autoencoder (VAE) to capture both temporal and amplitude patterns from each physiological measurement taken by the wearable devices for each night in a baseline. We then use statistical measures to distinguish outlier/abnormal nights within a baseline and consider them for removal.

The variational autoencoder is an unsupervised deep learning model with two parts: an encoder and a decoder. The encoder takes a feature set *x* and probabilistically transforms these features into latent variables *z*. The latent variables $z={Encoder}(x) \sim q({z|x})$ are the representation of the nightly physiological measurements in a reduced space of *R*^*d*^ where *d* is the dimension size. The decoder then uses the latent variables and reconstructs it to the original signal $x={Decoder}(z) \sim p({x|z})$. VAE can also control the distribution of the latent space through optimizing the reconstruction loss *L*_*rec*_ and the KL divergence loss *L*_*KL*_^42^. In this work, the decoder portion of the VAE is only used for training the latent space on the physiological measurements and there is no generative application utilizing the decoder. We opted to use a variational autoencoder over a normal autoencoder for the ability to control the distribution of the latent space. This manipulation of the encoded space allows us to control the variability of the encoded wearable data which results in better probabilistic selection of anomalous nights within the latent space.

To simplify the model architecture of the VAE, we opted to input only quantile information of each night in a baseline. Therefore ${z}^{m}={Encoder}({x}^{m}) \sim q({z}^{m}|{x}^{m})$ where ${x}^{m}=\{{q}_{i}^{m}{|i}=\mathrm{5,10},...95\}$ where ${q}_{i}^{m}$ is the *i*^*th*^ quantile of a data signal and *m* is the physiological measurement type, i.e., HR, RMSSD, etc. We also use only four key physiological measurements – heart rate, heart rate variability, respiration rate, and skin temperature – as input to the VAE model. This was empirically chosen after iterative investigation showed that these measurements were the most prominent in detecting physiological events.

Furthermore, we separate the pattern identification model into four separate VAEs, each trained on one of the key physiological measurements. This is to divide pattern identification into each physiological measurement within a baseline window and provide an opportunity to identify which category a night might be anomalous in. The full VAE model architecture can be found in Supplementary Note 8.

Candidacy for outliers is chosen based on the latent variables and their distance from one another. The main idea is that latent variables within a baseline night should highly represent each other and therefore be closer together in space. If an outlier/abnormal night is present in the baseline, the latent variables should reflect that by being a considerable distance away from the other nights’ latent variables. We calculate their distances by taking the mean pairwise Euclidean distance of each night’s latent variables from each other. This is calculated by the following equation:

2 $${d}_{i}^{m}=\frac{{\sum }_{j}^{n}\sqrt{{\left({z}_{i}^{m}-{z}_{j}^{m}\right)}^{2}}}{n}$$

where ${d}_{i}^{m}$ is the mean pairwise Euclidean distance of night *i* in the baseline window for measurement *m*, ${z}_{i}^{m}$ is the latent variable of night *i*, ${z}_{j}^{m}$ is the latent variables of other nights in the baseline window, and *n* is the total number of nights in the baseline window. Based on these distance metrics, we then use statistical methods to classify possible outliers. From this, we chose two methods to consider, standard deviation or interquartile range (IQR). Using standard deviation, we Z score transform the distances ${d}_{i}^{m}$ for each night and use a decision threshold *α* to decide whether a night falls outside of the threshold. If they do for a specific measurement, then that night is marked as a candidate for removal. For IQR, the flow is the same except for the criteria for classifying outliers. Using IQR, outliers are classified as

3 $${d}_{i}^{m}\,\ge\, {q}_{75}({d}^{m})+{IQ}{R}^{m}* \alpha$$

or

4 $${d}_{i}^{m}\,\le\, {q}_{25}\left({d}^{m}\right)-{IQ}{R}^{m}* \alpha$$

where ${IQR}=({q}_{75}({d}^{m})-{q}_{25}({d}^{m}))$, *α* is a specific scale, usually 1.5, and ${q}_{75}({d}^{m})$ and ${q}_{25}({d}^{m})$ are the 75th and 25th quantiles of the mean pairwise distances of the nights in the baseline window for measurement *m*. Mean pairwise distances falling outside of this range would then be marked as a candidate for removal.

A night being marked as a candidate for removal is only discarded if multiple measurements for the night within the baseline window also marked the night for removal. For example, for a specific subject with a 14-night baseline window, if a night is marked for removal within the HR, RMSSD, and Temperature models, then that night would successfully be removed from the baseline, reducing the number of nights in the baseline window to 13. However, if a night only was marked for removal from one measurement such as HR, then the night would stay in the baseline. This is to ensure that abnormalities in baselines do not occur within just one physiological parameter. Removing outlier nights with only one physiological parameter indicating abnormal behavior can result in other physiological features from other measurements being affected negatively. For example, one night within a baseline may have abnormal respiration rate behavior, but their skin temperature, heart rate, and heart rate variability show normal or even optimal behavior. Removing this night would affect the features extracted from temperature, HR, and HRV due to the removal of an optimal measurement from the baseline. Therefore, a consensus across multiple measurements for removal of a data point from the baseline ensures that abnormalities are confined to correlated physiological behaviors that affect multiple physiological systems in the body. Since four measurements were used for determining outliers, a consensus agreement between three measurements was logically used as the criteria to fully remove a night from a baseline window.

After removing outlier nights from the baseline window, Z-score transformation was then executed for segment data *D*_*i*_ with the new baseline window. This brings forth two datasets for symptom and infection classification to be trained and tested on: a control data and a baseline corrected data. As a precaution for data preservation and availability, the removal of nights from the baseline window is limited to the minimum number of nights needed for a valid baseline window. In the control data, this was set to 7 days minimum. Therefore, the baseline correction algorithm can eliminate nights from the baseline window until it reaches a minimum of 7 days in the baseline. However, in practice, removed outliers range from zero nights to up to three nights normally during testing in this study.

Baseline correction occurs during feature value transformation (baseline calculation) for each day of data captured by the wearables. After each day of captured data, the baseline window is extracted from past data, baseline correction is applied within the window, and the corrected baseline window is used for feature value transformation. It is important to note that baseline correction only requires data within the rolling baseline window to determine anomalies and does not utilize any information outside this window.

### Label correction via probability voting

The label correction algorithm presented in this work has been denoted as probability voting (PV); a method in which the temporal relationship between samples and the well-represented negative feature space is leveraged to efficiently and effectively correct symptom labels. PV takes windowed user data as an input and outputs a corrected label matrix for each input sample, defined as

5 $${w}_{\{i,i+1,\ldots ,i+n-1\}}^{p}{\rm{;}}{w}_{\{j,j+1,\ldots ,j+n-1\}}^{n}$$

where ${w}_{\{i,i+1,\ldots ,i+n-1\}}^{p}$ is a positive window containing samples *i, i* + *1, …, i* + *n-1*, with *n* being the number of samples in the window. These samples occur in chronological order such that *t*_*i*_ *< t*_*i+1*_ with all samples within the window belonging to the same user. Similarly, ${w}_{\{j,j+1,\ldots ,j+n-1\}}^{n}$ is a negative window with the same properties. Positive and negative windows differ in that at least one sample a positive window must have a reported label of 1, whereas all samples in a negative window must have a reported label of 0. Positive windows are centered around a positive report such that there are two nights before and two nights after the original report within the window. Given the nature of the dataset, the number of negative windows is substantially higher than the number of positive windows.

Each sample *i* has a reported label ${l}_{i}^{0}$ and features *f*_*i*_ such that

6 $${l}_{i}^{0}\in \{0,1\}{\rm{;}}{f}_{i}\in {{\mathbb{R}}}^{N}$$

where *N* is the number of features in the input set and a label of *0* or *1* correspond to a negative or positive label, respectively.

Probability Voting works by allowing samples within the training set to “vote” on other samples within the same training set to arrive at a conclusion as to what should be considered “negative” and “positive.” The process is divided into voting rounds, where a round is completed once each candidate sample has been voted on once. A single vote is defined as

7 $$\begin{array}{c}{v}_{i}^{k,j}={H}_{k,j}\left({f}_{i}\right)\\ {H}_{k,j}\left(x\right)\in {[0,1]}^{2}\end{array}$$

where ${v}_{i}^{k,j}$ is the jth vote for round k on sample *i* and ${H}_{k,j}\left({f}_{i}\right)$ is the opinion formed by the other samples in the training set. In this study, this opinion takes the form of a classifier that outputs a probability matrix corresponding to the likelihood of sample *i* being positive. To perform a vote, data from a subset of positive windows are selected as candidates while all remaining training windows (both positive and negative) are used to form an opinion and vote on each sample in the candidate windows. Once the voting round is complete, the votes are processed into labels according to the specified voting method.

Voting rounds may be completed once or iteratively, depending on the application. Each voting round will yield a noise matrix for each sample such that

8 $${l}_{i}^{0}\in \{0,1\}{\rm{;}}{f}_{i}\in {{\mathbb{R}}}^{N}$$

where ${l}_{i}^{k}$ is the label for sample *i* after voting round *k*. These labels are used for voting in round k + 1 unless all voting rounds have concluded in which they become the algorithm’s output.

While the PV algorithm is primarily focused on correction of sample labels within positive windows, the negative windows should also be addressed as they are vital to making intelligent decisions during the voting process. False negative samples present in the negative window set introduce the risk of incorrect opinions being formed, and thus removing these false positives from the training set should be a priority prior to starting the voting process.

The approach taken in this work is to perform one round of voting on negative windows prior to positive window voting rounds. However, rather than correcting the sample labels in negative windows (e.g. correct some negative nights to positive night within a negative window), nights with high probability of a symptom are simply excluded from the voting process. This avoids making assumptions about the presence of positive samples in negative windows and rather avoids suspect samples.

While a classifier may give label probabilities when voting, there are various ways one might approach handling the output matrix. There are 2 methods that have been developed and analyzed in this work: (1) threshold, and (2) maximum.

Threshold voting is the purest form of vote processing as it simply assigns sample labels based on whether or not they are above or below a specified threshold. Thus, after voting round k,

9 $${l}_{i}^{k}=\left\{\begin{array}{c}1,{{v}_{i}^{k,j}}_{2}\ge {threshold}\\ 0,{{v}_{i}^{k,j}}_{2} <\, {threshold}\end{array}\right.$$

where ${{v}_{i}^{k,j}}_{2}$ is the second element of the probability matrix output by the voter for sample *i* for the *jth* vote of round *k* (i.e. the probability of a positive label for sample *i*). Threshold voting allows the voting process to drop windows completely from training and add multiple nights per window. As seen in the results, this can be useful when the objective is easier to detect (e.g., fever).

Maximum voting works by only assigning a positive label to the night in a given window with the maximum probability of positive.

10 $${l}_{i}^{k}=\left\{\begin{array}{c}1,{{v}_{i}^{k,j}}_{2}={window\; maximum}\\ 0,{{v}_{i}^{k,j}}_{2}\,\ne\, {window\; maximum}\end{array}\right.$$

This approach restricts the voting process to only assign one positive label per positive window, without the ability to drop windows from training. This method is effective when the objective is hard to detect (e.g., shortness of breath).

While probability voting can label any sample within a positive window positive, regardless of when it occurs, it makes sense to bias towards nights closer to the report time to leverage the information we were given by each user. To achieve this, an additional parameter named the *trust score* is added to the PV algorithm. This trust score acts as a penalizer that discourages the voter from selecting samples that are farther away from the original report time. The trust score is defined as follows:

11 $${\hat{v}}_{i}^{k,j}={v}_{i}^{k,j}* {e}^{-c* \Delta t+c/2}$$

where ${\hat{v}}_{i}^{k,j}$ is the updated voter probability assigned to sample *i* for vote *j* during round *k*. Given a time-from-report ∆*t*, a higher trust score *c* will penalize sample *i* more, and therefore put more “trust” in the original report time. The second term in the exponential $c/2$ also promotes the original report time as *∆t =* 0 for this case. Therefore, as *c* increases, the labels produced by the PV algorithm approach the original report labels:

12 $${l}_{i}^{k}\mathop{\to }\limits_{c\to \infty }{l}_{i}^{0}$$

The full algorithm can be viewed in Supplementary Fig. 11.

### Classification machine learning model

We use a gradient boosted decision tree model – XGBoost^23^ – for symptom detection and COVID-19 prediction. This classifier takes as input the statistical features extracted during feature extraction of a night’s data and outputs the probability of a symptom or of infection for that night.

For the training phase, nightly data from all negative evaluation windows from all training subjects are used to form the negative training set while only positively labeled nights within all positive evaluation windows are used as the positive training set – negative labels within the positive evaluation windows are left out. This is to expose the model to the closest day to the symptom/diagnosis that possibly demonstrates the strongest physiological response while also removing any nights that had residual physiological responses but are marked as negative nights.

For the testing phase, each evaluation window is treated as a single sample. To predict an event for a window, each night within that window is fed into the classifier and given a probability of that event taking place. The subsequent event probability of that evaluation window is the maximum probability given to any of the contained nights.

### Performance evaluation

Due to the class imbalance amongst all symptomatic and COVID-19 cases, sample weights were applied accordingly during the training of each classifier to achieve a balanced loss across classes. To accurately represent improvements of the correction techniques introduced in this work, models were assessed across a range of number of estimators from 5 to 200, max depths from 2 to 8, trust scores of 0.0, 0.1, 0.2, 0.3, 0.4, and 0.5, and voting method/threshold pairs of *maximum*/0.0, and *threshold*/0.5, totaling 576 configurations. 5-fold cross-validation was implemented and the configurations with the best average were used. Results were obtained by selecting the best performers based on ROC AUC performance. This method was selected due to the low number of potentially noisy samples in a validation fold when using nested cross-validation. Thus, performances for 50 folds (10 5-fold splits) were averaged before selecting the configuration with the best performance. Supplementary Figs. 12–19 also demonstrate the general increase in performance across configurations, further improving confidence in our methods. These histograms show that even with random XGBoost configurations used, our methods can still boost performance as there is a shift towards positive improvement across most configurations used in the study.

To avoid data leakage, training and testing sets were separated by participant. These training and testing splits were performed across 10 random states yielding 10 unique 5-fold splits. This allows for an accurate representation of performance in a real-world environment, where models cannot use a priori knowledge of a participant’s labeled data to make predictions for that same participant.

In conjunction with using common metrics such as sensitivity, specificity, and ROC curves, we also utilize Precision-Recall (PR) curve and AUC to highlight the performance and improvements to the classifier model by our proposed correction techniques. Due to the nature of the study, negative, non-infectious/non-symptomatic days are more commonly expected than positive, symptomatic days. Improvements in the precise detection of positive cases are more clearly demonstrated in the PR curve as PR considers class imbalance. We evaluate the improvements in sensitivity and precision at set specificity of 75% and set recall of 75% to provide two analyses: 1) the improvement in true positive rate and 2) the improvement in true negative rate. The selection of 75% as the desired specificity in our evaluation metrics was based on a balance between the impracticality of low specificity and the unrealistic performance of high specificity. A low specificity rate introduces uncertainty in the usage of the model, whereas high specificity often results in not obtaining any relevant information. A similar rationale was applied in the determination of the threshold for recall.

We provide an analysis that examines the impact of label noise in the test set by applying the proposed label correction technique to the test set prior to evaluation. This correction is self-contained within the test set, i.e., no information outside the test set is used to influence the label correction process. To ensure that most positive evaluation windows are preserved, only the windows exhibiting the lowest probability of their respective event are converted to negative evaluation windows. This is done through threshold voting as described under the *Probability Voting* section, with the threshold being set at 0.05. This leads to only converting positive windows that show a less than 5% probability of containing their respective event. The correction of the test set should be precise, as poor label correction will lead to poor model performance during evaluation.

The overall pipeline of our methods can be viewed in Supplementary Fig. 20 and in-depth views of baseline correction and label correction using probability voting can be seen in Supplementary Figs. 21 and 22, respectively.

### Reporting summary

Further information on research design is available in the Nature Research Reporting Summary linked to this article.

### Supplementary information

Supplementary Information

Reporting Summary

## Data Availability

The datasets analyzed during the current study cannot be made available due to contractual framework.

## Code availability

The machine learning algorithms and the custom codes used for data visualization are available from the corresponding author upon reasonable request.
