# Supplementary material for: Early adverse physiological event detection using commercial wearables: challenges and opportunities
Source: NPJ Digit Med. 2024 May 23;7:136. doi: 10.1038/s41746-024-01129-1 (PMC11116498; doi:10.1038/s41746-024-01129-1)
Supplement: Supplementary file 1 — Supplementary Information [file 41746_2024_1129_MOESM1_ESM.docx]

SUPPLEMENTARY INFORMATION

for

Early Adverse Physiological Event Detection using Commercial Wearables: Challenges and Opportunities

Jesse Phipps^*1^, Bryant Passage^*1^, Kaan Sel^2^, Jonathan Martinez^1^_­_, Milad Saadat^2^, Teddy Koker^3^,

Natalie Damaso^3^, Shakti Davis^3^, Jeffrey Palmer^3^, Kajal Claypool^3^, Christopher Kiley^4^,
Roderic I. Pettigrew^5^, Roozbeh Jafari^1,2,3,5,6^

^1^Department of Computer Science and Engineering, Texas A&M University, College Station, TX, USA

^2^Department of Electrical and Computer Engineering, Texas A&M University, College Station, TX, USA

^3^Lincoln Laboratory, Massachusetts Institute of Technology, Lexington, MA, USA

^4^Defense Threat Reduction Agency, Fort Belvoir, VA, USA

^5^School of Engineering Medicine, Texas A&M University, Houston, TX, USA

^6^Laboratory for Information and Decision Systems, Massachusetts Institute of Technology, Cambridge, MA, USA

*These authors contributed equally: Jesse Phipps, Bryant Passage

Corresponding author: [roozbeh.jafari@ll.mit.edu](mailto:rjafari@tamu.edu)

SUPPLEMENTARY NOTE 1. FURTHER ANALYSIS ON INHIBITING FACTORS FOR INFECTION PREDICTION

There are four challenges that inhibit the performance of infection prediction using COTS wearables: (1) heterogeneity among and within participants that make scaling detection algorithms to a general population less precise, (2) confounders that lead to incorrect assumptions regarding a participants healthy state (3) noise in the data at the sensor level that limits the sensitivity of detection algorithms, and (4) imprecision in self-reported labels that misrepresent the true data values associated with a given physiological event. We further analyze the effects of these challenges on user data and how they negatively impact prediction performance of machine learning models built for infection detection.

For patient heterogeneity, each participant in the cohort experiences unique resting heart rates behaviors. This can be seen in Supplementary Figure 1. Quantitatively, we can see a large distribution of nightly heart rates across the patients in this study. This inhibits the machine learning algorithm to learn generalizable relationships between the physiological responses of a participant experiencing COVID symptoms and participants in a normal resting state.

Eliminating daytime data and only using nocturnal data mitigates noise caused by participant daytime activities. However, there are still other factors that affect nighttime data as well. Undesired confounders include irregular activities that disrupt the normal resting state of a person. Examples of this include late caffeine/alcohol intake prior to sleeping, or even disrupted sleep schedules due to work. Supplementary Figure 2 shows how participants may experience irregular physiological responses throughout the week. While context is provided to the prediction model regarding what activity of daily living may have led to varying physiological responses from day-to-day, therefore, baseline correction aims to remove these irregular responses from the baseline calculations and improve the Z-score calculation used for training and predicting infections. Utilizing baseline correction can also help alleviate sensor and instrument noise on a night-to-night basis. Noise contaminating night-time data can embed erroneous signals which then get captured in baseline windows. With baseline correction, noise that creates anomalous data, similar to noise created by undesired confounders can be eliminated.

Imprecision of surveys is demonstrated in Figure 5, where physiological responses either do not properly align with symptom reports or are absent around the time of report. This leads to inaccurate representations in the data, where measurements from non-infected periods are labeled as infected and measurements from infected periods are labeled as non-infected. This therefore hinders the performance of detection models constructed from this inaccurately labeled data.”

SUPPLEMENTARY NOTE 2. FPR ON HEALTHY COHORT

To extend model performance to additional participants, an additional cohort of participants reporting only negative COVID-19 test results was derived. Healthy evaluation windows were then obtained from these participants, totaling 388 unique participants and 1364 windows. For this analysis, this new cohort will be denoted as the “healthy cohort,” whereas the cohort presented in the main manuscript will be denoted as the “positive COVID-19 cohort.” For each fold during validation, a threshold was selected to achieve a specificity of 95% on the validation fold from the positive COVID-19 cohort. The model trained for this fold then predicted across each window in the healthy cohort and used the threshold from validation to determine positive/negative predictions. This was repeated across 10 5-fold splits, yielding 50 total FPR values for the healthy cohort at 95% model specificity. Using these FPR values, we conducted a one-sample t-test with 49 degrees of freedom to test the null hypothesis that the FPR on the healthy cohort is 5%. The results are shown in Supplementary Table 1. As seen in the table, there is not enough evidence to suggest that the FPR on the windows in the healthy cohort differs from the FPR across the healthy windows in the positive COVID-19 cohort.

SUPPLEMENTARY NOTE 3. SYMPTOMATIC VS. ASYMPTOMATIC

Assessment of early detection performance on asymptomatic vs. symptomatic cases. Symptomatic cases were defined as any COVID-19 evaluation window that contained participant reports of any of the following symptoms: fever, shortness of breath, fatigue, sputum, cough, or loss of taste or smell. Asymptomatic cases were defined as those which had none of the symptomatic events reported in the evaluation window leading to a positive test result. There were a total of 78 asymptomatic cases and 63 symptomatic cases. It should be noted that evaluation windows in this study were for the task of early detection, which was defined as 24 hours before a positive test result. It was found that detection performance for asymptomatic cases improved from 0.60 ROC AUC to 0.63, with detection performance on symptomatic cases improving from 0.80 to 0.84 ROC AUC as seen in Supplementary Table 2. This indicates that asymptomatic cases are detected at a performance above random, which implies the value of wearable-based diagnostics where such asymptomatic cases would inherently not be captured by a symptom report-based detection approach.

SUPPLEMENTARY NOTE 4. IMPACT OF EARLY DETECTION OF COVID-19

Earlier detection of COVID-19 by 0.6 days can have a considerable impact to infection spread within a population. With the assumed exponential growth rate of infections such as COVID-19, without any controlled intervention, the virus can rapidly spread across a population, causing strain in resources used for treatment. In this study, we assume that our prediction model is used as an early warning device that allows its users to be informed of the possibility of infection and prepare for isolation and quarantine. While our model does not predict cases on an hourly basis but rather a daily basis, we can assume that the 0.6 day improvement can be split into half of the population receiving notification of COVID-19 positive case a day earlier. Detection of COVID-19 one day earlier can result in faster isolation by more than half of the cases that will receive a positive PCR test in the future. Furthermore, according to the viral load of COVID-19, there exists a brief, yet valuable time where PCR tests are able to detect COVID-19, however, the virus is not yet infectious, and adding an additional day of isolation allows for higher probability that the virus is not yet infectious prior to when participants take their PCR tests [1]. In theory, in the worst-case scenario, if all the positive detection cases prior to the enhanced earlier detection of COVID-19 were during the time the virus is infectious, and other healthy persons were infected prior to isolation, we can assume a full growth rate of $r$ to exist ($r$ varies between cohorts depending on multiple environmental and social conditions; we can assume $r$ = 0.2 according to Musa et. al for this analysis) within Liang’s eq. 3 ($N(t)=\frac{N_{max}}{1+\left( \frac{N_{max}}{N_{0}}-1 \right)e-rt}$)  where $N_{0}$ is the starting number of infections, $r$ is the growth rate, $t$ is time elapsed, and $N_{max}$ is the limit of $N(t)$ - where no new cases are observed [2]. However, assuming that half the positive cases received their COVID-19 notification a day earlier and started self-isolation, with the assumption that the virus is not yet infectious during this time, the transmission rate will be halved. This means approximately $\int_{0}^{t} \frac{N_{max}}{1+\left( \frac{N_{max}}{N_{0}}-1 \right)e^{-rt}}dt-\int_{0}^{t} \frac{N_{max}}{1+\left( \frac{N_{max}}{N_{0}}-1 \right)e^{-\frac{1}{2}rt}}dt=\int_{0}^{60} \frac{1369}{1+\left( \frac{1369}{100}-1 \right)e^{-0.2t}}dt-\int_{0}^{60} \frac{1369}{1+\left( \frac{1369}{100}-1 \right)e^{-0.1t}}dt\approx$17488 total cases, or 27.2% of cases will be averted in a span of 60 days (with a growth rate of 0.2 per day [3], starting number of infections $N_{0}=100$ and an $N_{max}=1369$, using Liang’s equation [2] and data from the Guangdong region [2], respectively). Although this depicts a worst-case scenario, it can be seen that a 0.6 day improvement in detection time can result in more than a quarter of positive cases being averted over the course of two months since an initial outbreak. Therefore, it is imperative that our model is able to predict COVID-19 as early as possible to avert as many cases as possible.

SUPPLEMENTARY NOTE 5. FEATURE VALUES OF FEVER AND ELEVATED TEMPERATURE

We further investigate the feature values that are input into the classifier model for temperature cases above 100.4$^{\circ}$F (fever) and temperature cases between 99.1$^{\circ}$F and 100.3$^{\circ}$F inclusive (elevated temperature) which can be seen in the bar plot in Supplementary Figure 4. From this plot, we can see the increased difficulty in detection of elevated temperature cases versus fever cases as Z-scores for elevated temperature cases are significantly lower in deviation. This accounts for the higher detection performance in fever over elevated temperature cases. Furthermore, our correction algorithms are less effective in fever cases due to the feature values being notably more pronounced for temperature cases above 100.4$^{\circ}$F, resulting in already high conventional performance. Conversely, the classifier detecting elevated temperature cases benefits more from our correction algorithms as the feature values for this temperature range are less distinct from features found in non-fever data.

SUPPLEMENTARY NOTE 6. BASELINE WINDOW ANALYSIS

We investigate the optimal number of days in the baseline window needed to achieve comparable performance in the detection of symptoms and prediction of COVID-19 diagnosis. We perform two analyses: 1) we analyze a range of absolute number of days to be used in each baseline window (e.g., a 7-day baseline window must have exactly 7 days of data represented in the window), and 2) we analyze a range of minimum number of days in a 14 day baseline window (e.g., a minimum of 3 days baseline window can have anywhere between 3 to 14 days of data represented in the window). For these analyses, we utilize the conventional framework with no data correction techniques applied on the data before training or testing. For the absolute number of days in baseline, after approximately 11-14 days in a baseline window, the increase in performance becomes marginal. However, larger number of days used in each baseline window also does not allow detection or prediction capabilities during early usage of a device (e.g., users that just started using a wearable with only 6 days of data collected would not be able to predict infections until after approximately one month of continuous usage in a 28 day baseline window requirement) which is seen in Supplementary Figure 5 as data availability rapidly decreases with increasing number of days required in each baseline window. However, requiring an absolute number of days in a baseline window to be present removes a large portion of predictable days as many individuals within the study rarely perfectly and continuously use the provided wearable devices with no gaps in their data. Therefore, we instead choose a default number of days in the baseline window and enforce a minimum to ensure data availability while also obtaining nominal model performance. For this analysis, we used a 14-day baseline with varying minimum number of days as a lower limit to the window. After a minimum of approximately 5-7 days in a 14-day baseline, the performance of the model begins to plateau. From these analyses, we settled on a 14-day baseline window with a minimum of 7 days within the window to ensure ample data availability while maintaining model performance.

We also incorporate a 7-day offset between a baseline window and the standardized prediction day to ensure that no possible onset of symptoms or physiological events are included in the baseline window (e.g., if we need to standardize/transform the features at $D_{0}$, we utilize $D_{-21}$ to $D_{-7}$ as the baseline window).

SUPPLEMENTARY NOTE 7. CORRELATION ANALYSIS OF COVARIATE TRAJECTORIES LEADING TO FEVER ONSET

To assess the temporal alignment of data between participants, we analyzed the correlations of covariate trajectories leading to fever onset. This was done by first obtaining multivariate time-series data across the 8 days leading to a sample labeled positive for fever. The four covariates selected for assessment were the 97.5 quantile of peripheral skin temperature, the 2.5 quantile of heart rate, the maximum respiration rate, and the minimum HRV RMSSD. This was done for each participant in a randomly selected training fold for fever detection, where there were 31 participants in the set. For example, if fever was originally reported by a participant on day t_0_, then the 8-day time-series trajectory for analysis would be from day t_0_-7 to t_0_, totaling 8 days. Since each data point represents one night of physiological measurements, this results in time-series vectors of size 1x8 for each participant (31 total). When label correction is applied to this training set, it would be expected that some 8-day vectors would shift in time. Using the previous example, if the day before the original fever report is labeled as positive for fever by the Probability Voting algorithm (day t_0_-1), we would then define the 8-day time-series vector for this participant from t_0_-8 to t_0_-1. After label correction is applied to the training set, we would expect the Pearson correlation coefficient between vectors to increase on average due to the better alignment of signals. With 31 participants in the set, we obtained $\frac{31\times(31-1)}{2}=465$ of these correlation coefficients comparing trajectories between all participants in the training set.

As expected, we observe an increase in correlations between participant trajectories after applying label correction to the training set as seen in both Supplementary Figure 6 and Supplementary Figure 7. This is most apparent in the correlation between peripheral skin temperature trajectories (Supplementary Figure 6b and Supplementary Figure 7b) where the average correlation between participant trajectories increased from 0.58 to 0.80 with label correction methods applied, which is expected considering the trajectories are aligned by fever events. This allows us to demonstrate the positive effects our label correction method has on the data, where temporal events are aligned between participants leading to improved physiological event representations in the dataset.

SUPPLEMENTARY NOTE 8. VARIATIONAL AUTOENCODER ARCHITECTURE

The VAE model’s encoder is constructed with 3 1D convolutional layers with varying kernel sizes and a stride of 2 for each convolutional layer. The kernel sizes are 5, 3, 3 from the outermost layer to the innermost CNN layer respectively. Large kernel sizes were chosen because the idea is to capture the overall general trend of the quantile distribution of the physiological signals. Each convolutional layer is followed by a batch normalization and ReLU activation layer. A fully connected layer is then added to the encoder before connecting to two more fully connected layers which will be used to compute the mean and variance used for sampling the latent variable $z$ and compute the KL divergence loss. The decoder is simply the reverse of the encoder with a fully connected layer as input layer to the latent variables followed by 31D convolutional transpose layers after. The resulting $\bar{x}$should be a similar reconstruction to the original $x$ input.

Furthermore, we utilize four VAEs, each on a separate measurement $m$ to find outliers based on a specific physiological measurement within a baseline window. This allows for added granularity in deciding for full removal of a night from a baseline window.

| Supplementary Table 1: FPR on Healthy Cohort at 95% Specificity | | | | | | |
| --- | --- | --- | --- | --- | --- | --- |
|  | Conventional |  | BC |  | BC + LC |  |
| Mean | 5.00% |  | 5.70% |  | 4.80% |  |
| STD | 3.50% |  | 4.00% |  | 2.30% |  |
| p-value* | 0.93 |  | 0.24 |  | 0.48 |  |
| *Derived from a one-sample t-test with μ=0.05 (5%) being the null hypothesis | | | | | |  |

SUPPLEMENTARY TABLE 1. FPR on Healthy Cohort at 95% Specificity

SUPPLEMENTARY TABLE 2. SYMPTOMATIC VS. ASYMPTOMATIC COVID-19 DETECTION PERFORMANCE

| **Supplementary Table 2.** Symptomatic vs. Asymptomatic COVID-19 Detection Performance | | |
| --- | --- | --- |
|  | **Before Data Correction** | **After Data Correction** |
| **Symptomatic (n=63)** | 0.80 | 0.84 |
| **Asymptomatic (n=78)** | 0.60 | 0.63 |

SUPPLEMENTARY TABLE 3. DETECTION PERFORMANCE ACROSS OFF-THE-SHELF METHODS FOR NOISY LABEL LEARNING


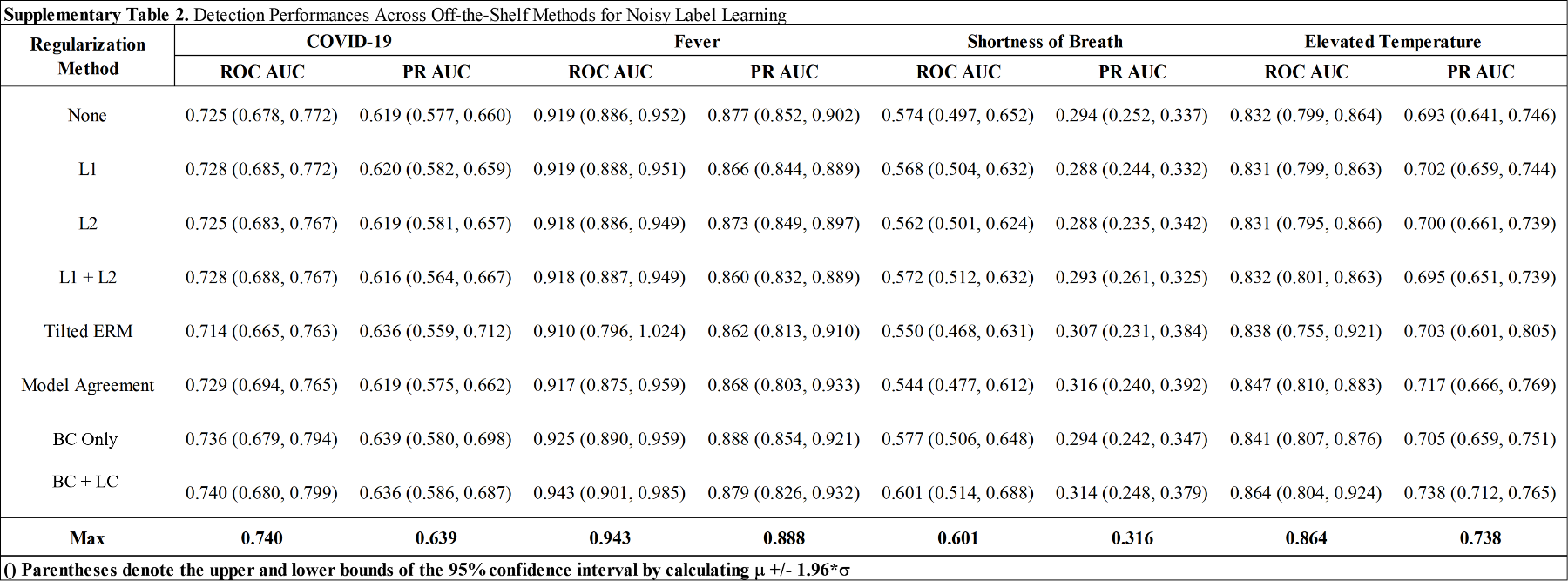


SUPPLEMENTARY TABLE 4. Off-the-Shelf Method Hyperparameters

| **Supplementary Table 4:** Off-the-Shelf Method Hyperparameters | | | |
| --- | --- | --- | --- |
| L1 | alpha = 0.5 | reg_alpha = 0.2 |  |
| L2 | reg_lambda = 0.2 |  |  |
| L1+L2 | alpha = 0.5 | reg_alpha = 0.2 | reg_lambda = 0.2 |
| TiltedERM | q = 0.5 |  |  |
| Model Agreement | agreement constant = 0.1 |  |  |

SUPPLEMENTARY FIGURE 1.


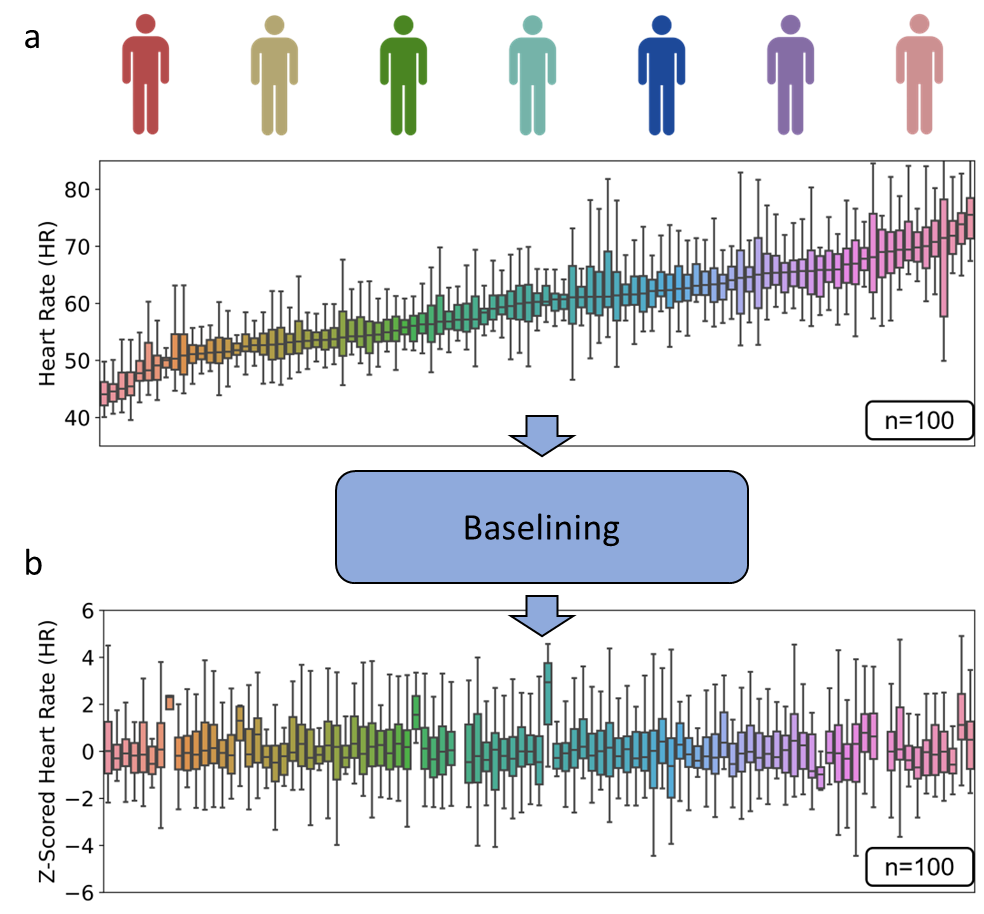


**Supplementary Figure 1. Boxplots of 100 randomly selected users and their heart rate data.** **a**, raw heart rate data and **b**, normalized heart rate data. Without any normalization techniques, high variances in physiological information between participants can result in overly complex models, or models that are not generalizable to different cohorts. Baselining alleviates this complication by centering or normalizing each participant’s heart rate with respect to their baseline window. Center Line: median, bounds: Q75-Q25, whiskers: $\geq IQR*1.5$

SUPPLEMENTARY FIGURE 2.


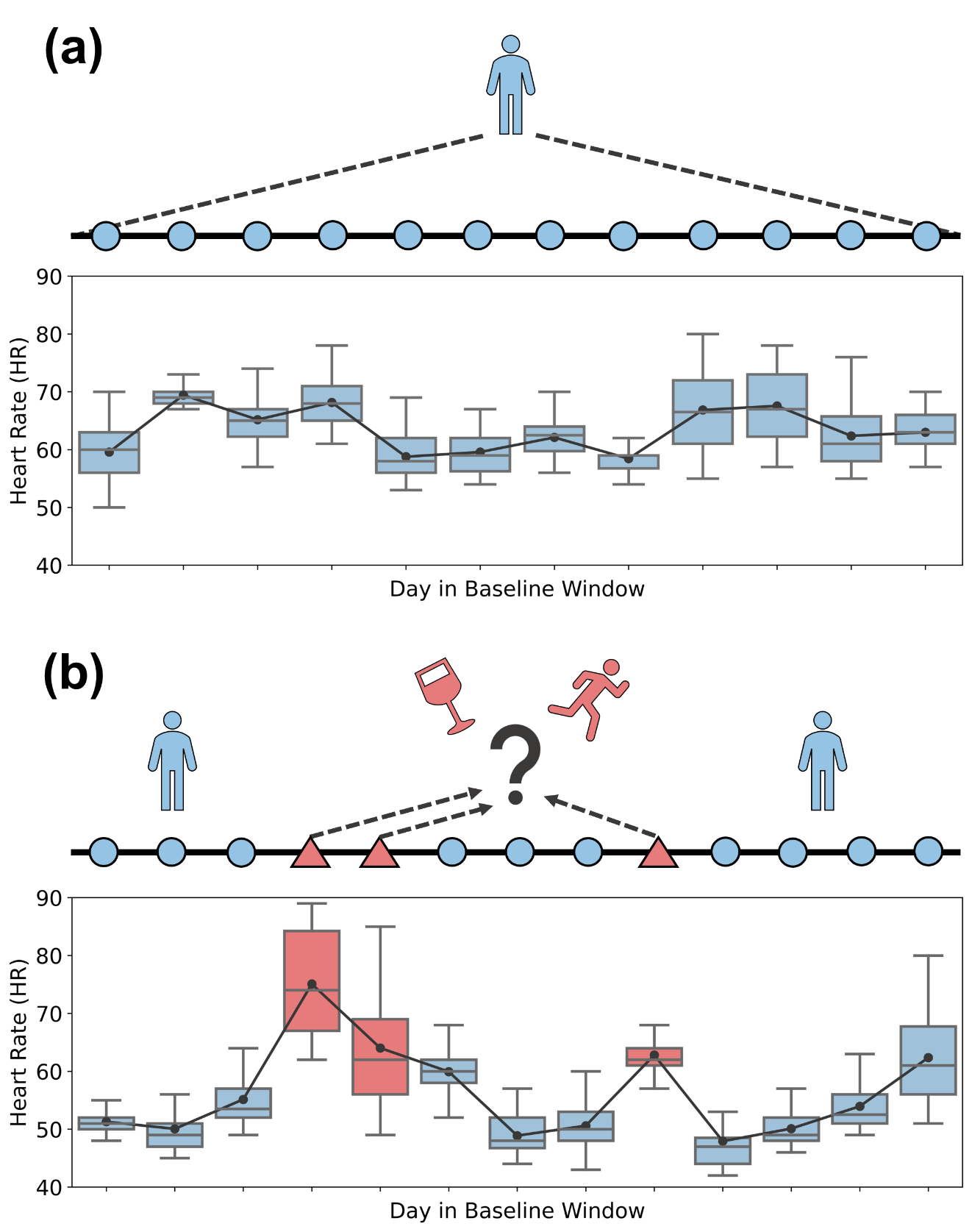


**Supplementary Figure 2.** **Example of the distribution of HR each day in a baseline window.** **a**, each day has a consistent distribution of HR over time with respect to each other versus **b**, certain days appear to be anomalous to other days within the baseline window.

Center Line: median, bounds: Q75-Q25, whiskers: $\geq IQR*1.5$

SUPPLEMENTARY FIGURE 3.


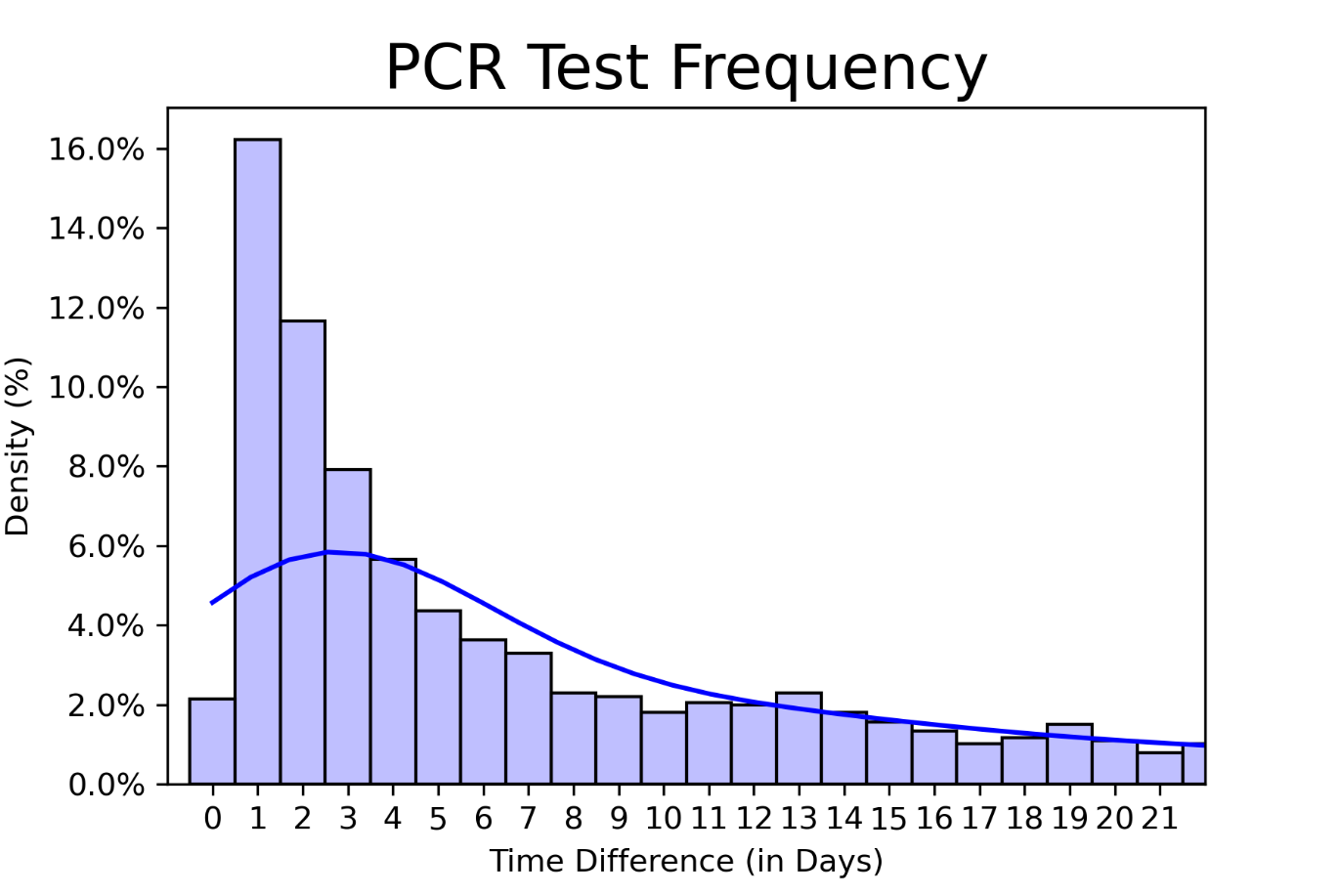


**Supplementary Figure 3. Frequency of PCR tests administered during the study.** As shown above, PCR tests were not triggered by symptoms, but rather administered periodically to preserve the study for detecting early physiological responses to COVID-19. The histogram also only contains time difference for each user up to their first positive COVID test to avoid frequent retest that might skew the distribution right.

SUPPLEMENTARY FIGURE 4.


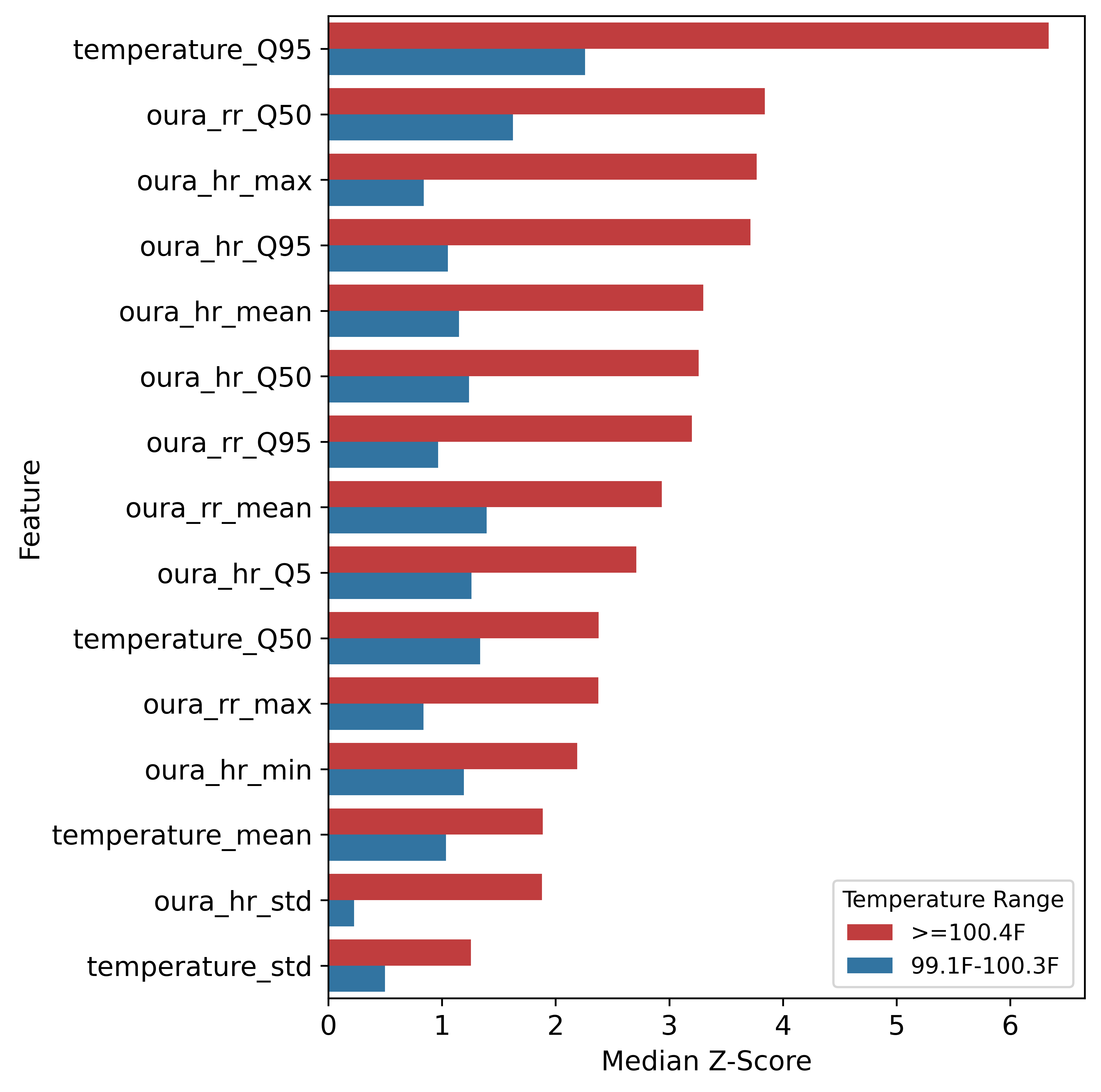


**Supplementary Figure 4. Median feature Z-score values of the top contributing features to the XGBoost classifier model.** Feature values for fever >=100.4$^{\circ}$F are in red and elevated temperature cases between 99.1$^{\circ}$F and 100.3$^{\circ}$F are in blue.

SUPPLEMENTARY FIGURE 5.


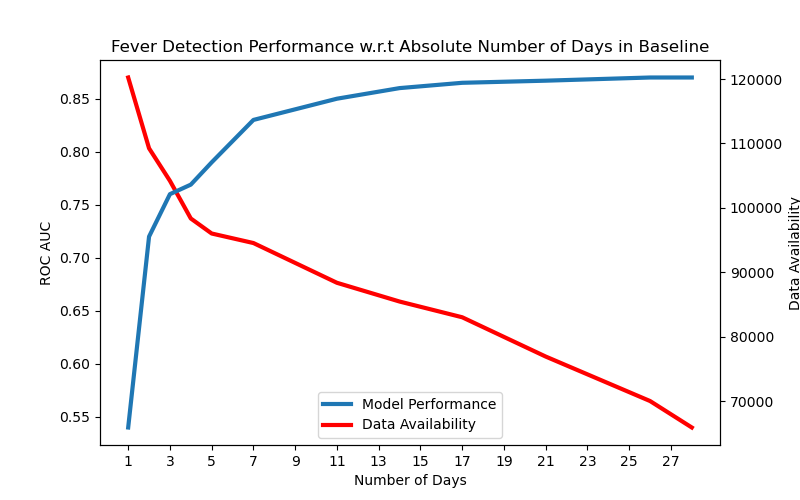

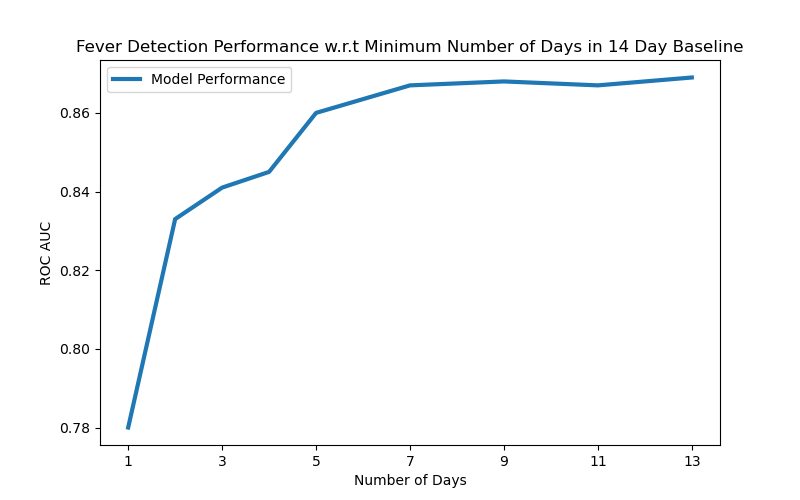


b

a

**Supplementary Figure 5. Model performance across different number of days used in the baseline window and the minimum number of days within a 14-day baseline window.** As the absolute number of days required in the baseline window increases, model performance increases as noise is mitigated by more samples for standardization **a**. However, after approximately 14 days, model performance improvement starts to marginalize, and data availability continues to rapidly decrease. When enforcing a minimum number of days in the baseline **b**, a smaller number of days are needed within a targeted 14-day baseline window. Therefore, this study used a target of 14-day baseline windows with a minimum of 7 days to maintain data availability and model performance.

SUPPLEMENTARY FIGURE 6.

**
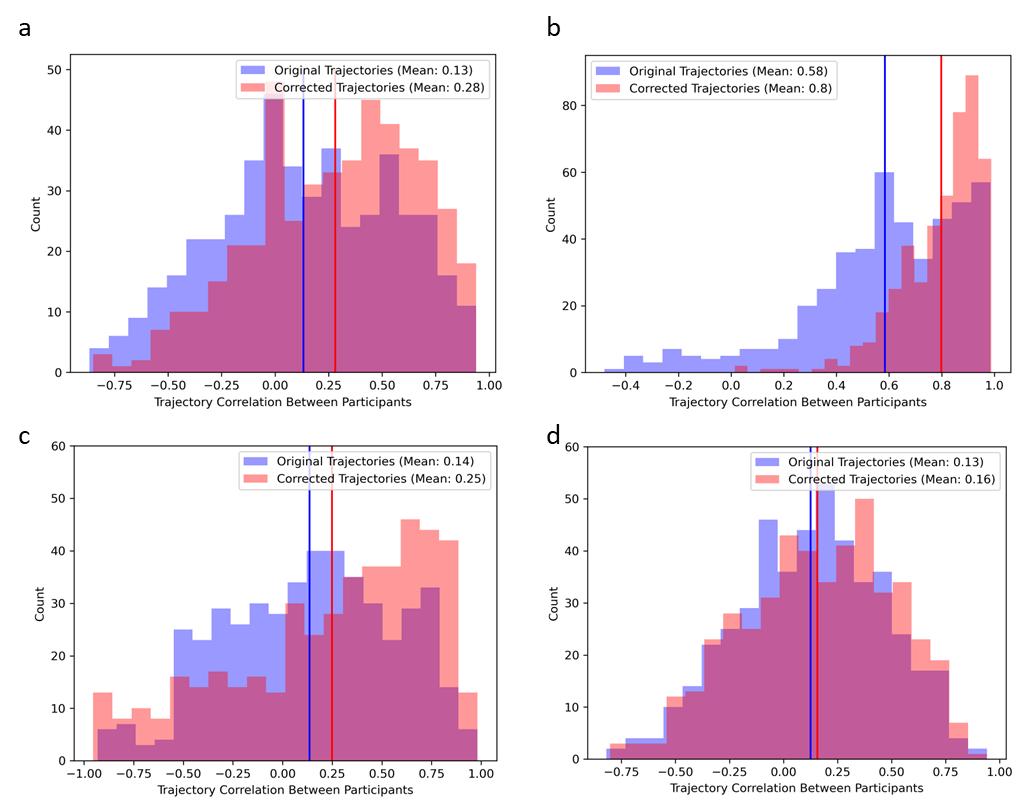
**

**Supplementary Figure 6. Correlation of 8-day biometrics leading up to the first labeled fever sample across 31 feverish participants in a randomly selected training fold before and after label correction techniques were applied to the training set.** Blue and red colors indicate before and after label correction, respectively. The shown biometrics are; (a) heart rate, (b) temperature, (c) respiration rate, and (d) HRV RMSSD trajectories. It is observed that correlations between temperature trajectories increase significantly, which would be expected when aligning fever onsets.

SUPPLEMENTARY FIGURE 7.


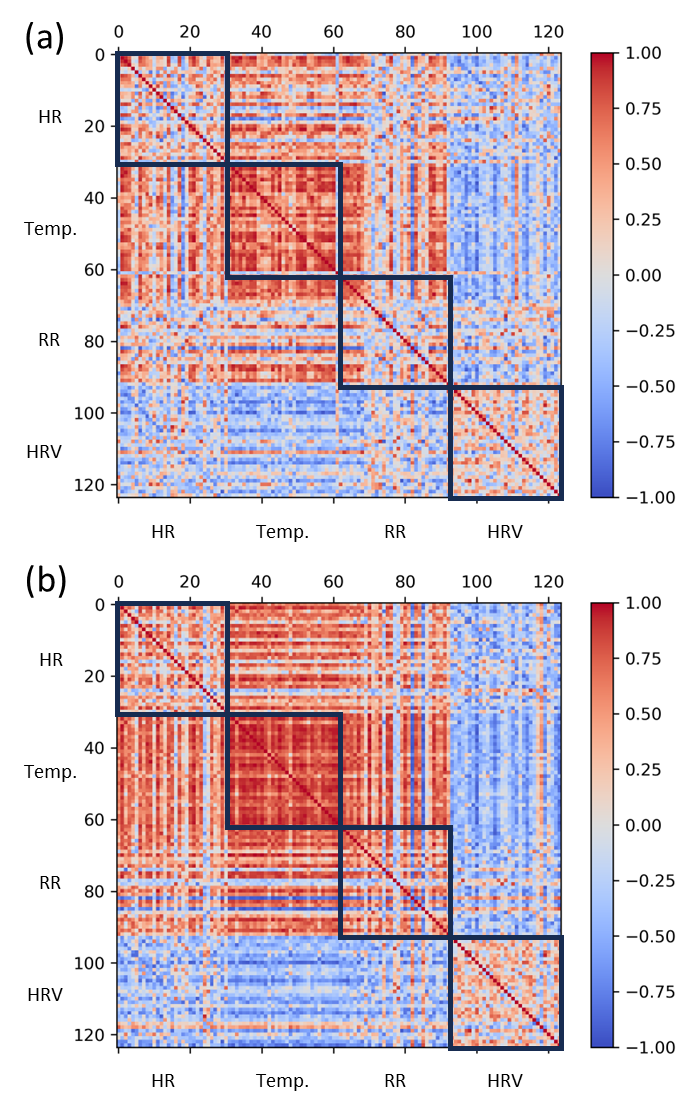


**Supplementary Figure 7. Correlation matrix for covariate trajectories. (a)** Correlation matrix before label correction was applied to a randomly selected training set for fever detection. **(b)** Correlation matrix after label correction was applied. Each row/column represents a single trajectory for a participant. With 31 participants in the set and 4 trajectories per participants (HR, Skin Temperature, RR, and HRV), the matrix has a size of 124x124.

SUPPLEMENTARY FIGURE 8.

**
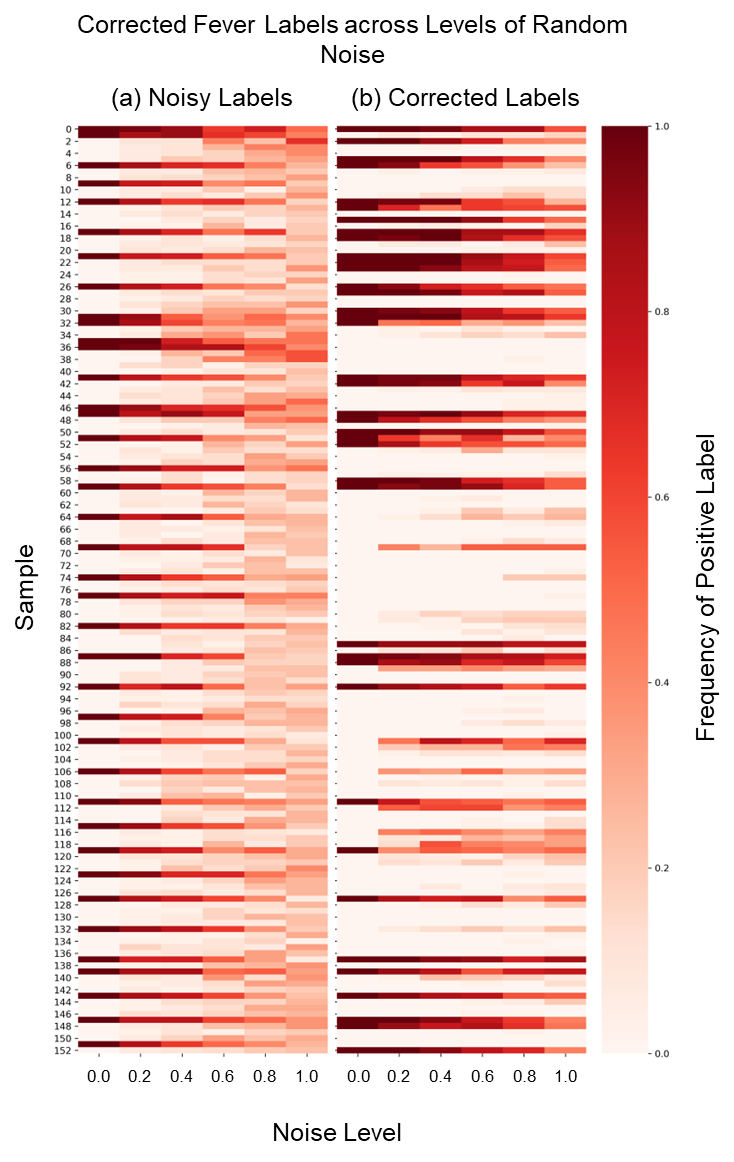
**

**Supplementary Figure 8. Frequency of positive fever labels.** **(a)** Within noisy and **(b)** corrected label sets for a single training fold, where rows denote a single sample within a positive window (n=152). Noise was applied by assigning a random zero-to-two-day shift to a given label. For example, a fever label reported on a Wednesday would be shifted to any day between Monday and Friday of that same week. The noise level signifies the ratio of evaluation windows affected by the synthetically injected label noise. If there are 100 evaluation windows in the training set, then a noise level of 0.4 would lead to 40 of these training windows being affected by the synthetically injected noise. The noisy label set (a) is then used in the probability voting algorithm to achieve the corrected label set (b). At a noise level of 0.0, the originally reported labels are seen in the noisy set. As seen in the figure, the labels assigned to samples in the corrected set are more resistant to the noise present in the noisy label set, indicated by darker lines at higher noise levels.

SUPPLEMENTARY FIGURE 9.


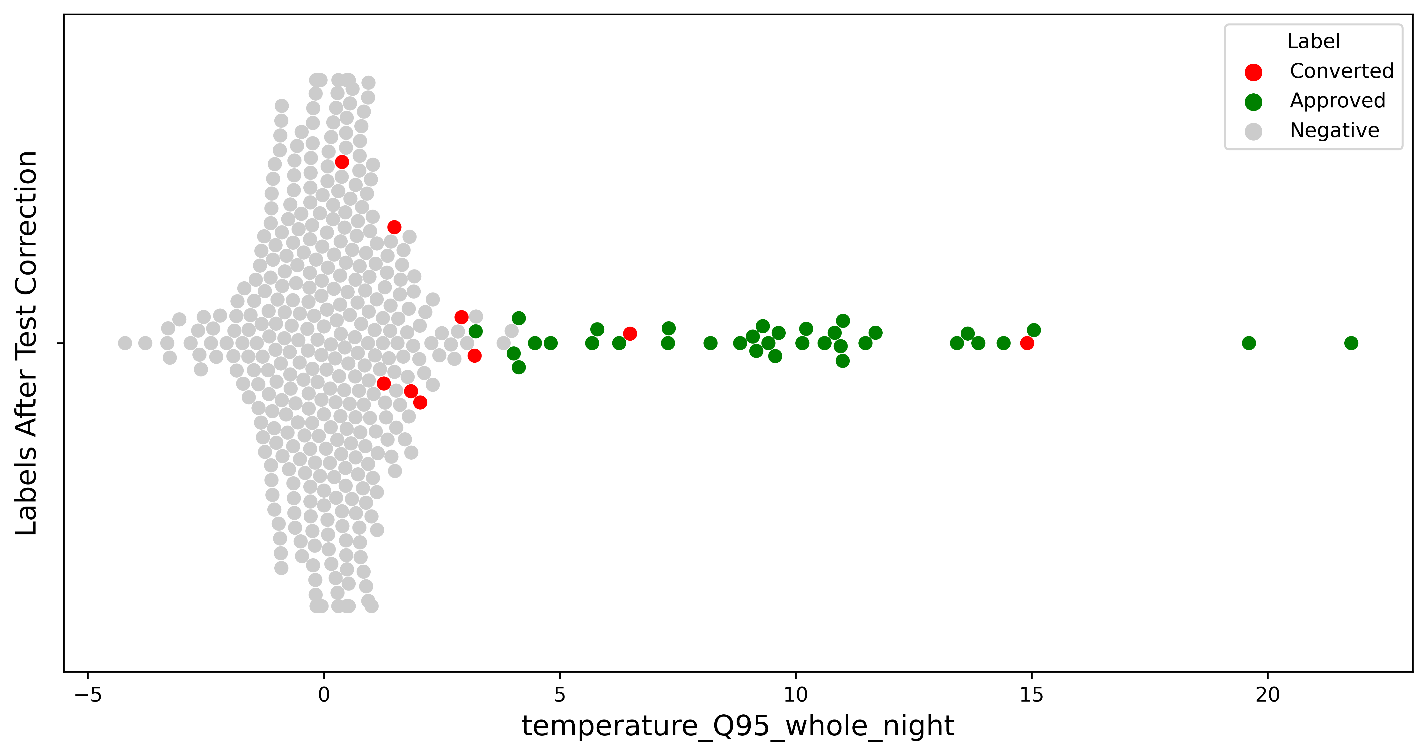
**Supplementary Figure 9.** **Swarm plot of label correction on the test set for fever.** Gray points represent negative labels of fever while colored points represent original positive labels reported by participants. Of the colored points, green points are labels that are reconfirmed as positive labels after label correction while red points are converted to negative labels after label correction. This shows that converted labels have feature values that more closely resemble negative labels, further reinforcing the reliability of the results from evaluations on the corrected test set. It should be noted that while there are some converted points that are closer to the approved labels than negative labels, this plot is specific to the most contributing feature for fever detection (Q95 of temperature). Other features, such as HR and HRV, may still influence the label correction process.

SUPPLEMENTARY FIGURE 10.

**
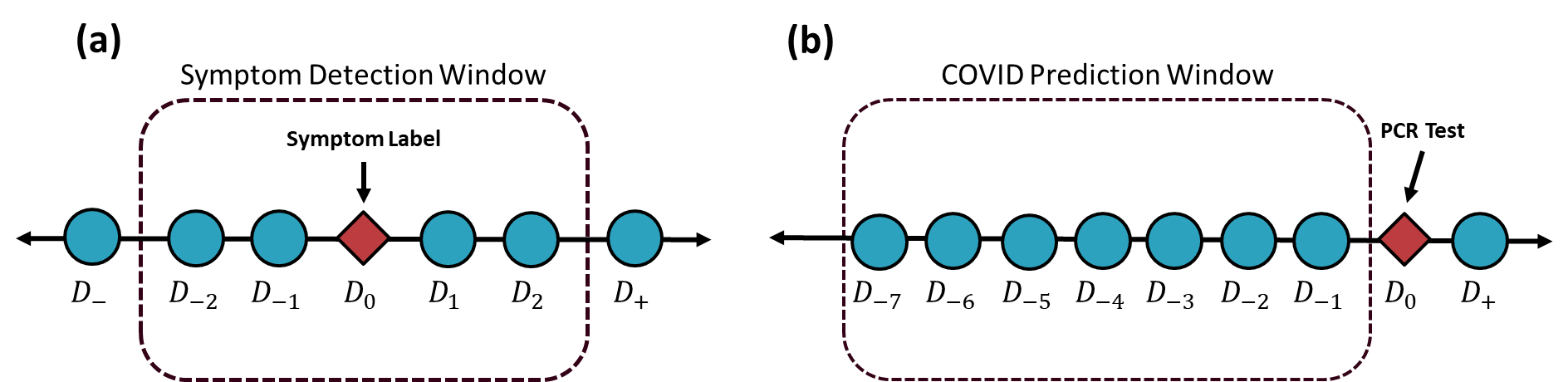
**

**Supplementary Figure 10.** **Evaluation windows used during testing and performance characterization of the XGBoost model.** **a**, shows how symptoms, i.e. fever and shortness of breath, are detected. The window includes 2 days prior to and after the symptom label to account for labeling errors by the participants. Therefore, if the model generates a positive probability greater than 0.5 anywhere between $D_{-2}$ and $D_{+2}$, then this particular window is considered a positive prediction. Likewise, **b,** shows the COVID-19 prediction window. The main difference is that for COVID, the window is before the PCR test label to allow the model to predict COVID between $D_{-7}$ and $D_{-1}$. Therefore, if any of those days in the window generate a prediction probability >0.5, then this would count as one positive prediction. It is important to note that these windows are NOT used during the training phase of the model. Furthermore, it should also be noted that negative COVID-19 windows do not necessarily precede a negative PCR test, but are instead 7-day healthy windows without any reported events in them.

SUPPLEMENTARY FIGURE 11.

| **ALGORITHM 1: Probability Voting** | | | | | | | |  | |
| --- | --- | --- | --- | --- | --- | --- | --- | --- | --- |
|  | INPUT: Positive and negative training windows  OUTPUT: Corrected label matrix for training samples | | | | | | |  | |
| **1** | positive-windows ← [$w_{\{i, i+1, \ldots,i+n-1\}}^{p}, w_{\{j, j+1, \ldots,j+n-1\}}^{p}, \ldots$] | | | | | | |  | |
| **2** | negative-windows ← [$w_{\{k, k+1, \ldots,k+n-1\}}^{n}, w_{\{l, l+1, \ldots,l+n-1\}}^{n}, \ldots$] | | | | | | |  | |
| **3** | negative-windows-cleaned ← clean-negatives(negative-windows) | | | | | | |  | |
| **4** | **while** (k < rounds) do | | | | | | |  | |
| **5** |  | C ← $\left[ \begin{matrix} w_{\{i, i+1, \ldots,i+n-1\}}^{p},w_{\left\{ j, j+1, \ldots,j+n-1 \right\}, \ldots}^{p} \\ \ldots\\ w_{\{m, m+1, \ldots,m+n-1\}}^{p}, w_{\{N, N+1, \ldots,N+n-1\}}^{p} \end{matrix} \right]$// where C is a $Q\times R$ matrix | | | | | |  | |
| **6** |  | j ← 0 | | | | | |  | |
| **7** |  | **while** (j $\leq$ Q) do | | | | | |  | |
| **8** |  |  | candidate-windows ←positive-windows $\in\boldsymbol{C}_{\boldsymbol{j}, *}$ | | | | |  | |
| **9** |  |  | positive--voters←positive-windows $\notin\boldsymbol{C}_{\boldsymbol{j}, *}$ | | | | |  | |
| **10** |  |  | obtain $H_{k,j}$ through positive-voters and negative-windows | | | | |  | |
| **11** |  |  | **for** window in candidate-windows do | | | | |  | |
| **12** |  |  |  | **for** i in window do | | | | | |
| **13** |  |  |  |  | $v_{i}^{k, j}\leftarrow H_{k,j}(x_{i})$ // vote on sample *i* using its features | | | | |
| **14** |  |  |  | **end** | | | | | |
| **15** |  |  |  | **for** i in window do | | | | | |
| **16** |  |  |  | $Y_{i}\leftarrow voting\_selection\_method(v_{i}^{k, j})$ // convert votes into labels | | | | | |
| **17** |  |  |  | **end** | | | | | |
| **18** |  |  | **end** | | | | |  | |
| **19** |  | **end** | | | | |  | | |
| **20** | **end** | | | | |  | | |  |

**Supplementary Figure 11. Probability voting algorithm.** Positive and negative windows are first constructed from the time series data, denoted as $w_{\{i, i+1, \ldots,i+n-1\}}^{p}$ and $w_{\{k, k+1, \ldots,k+n-1\}}^{n}$, respectively, where the window $w$ has either a positive or negative denotation and consists of samples $i, i+1, \ldots,i+n-1$. Negative windows can optionally be preprocessed before the voting process begins. For each voting round k, a $Q\times R$ matrix of candidate groupings are initiated, constituting matric *C*, where the matrix rows are candidate groupings of positive windows. For *Q* votes, the candidate group and positive voters are selected from the matrix *C*. The positive voters are combined with the negative windows to train the classifier $H_{k,j}$ for round *k*, vote *j*. It should be noted that only the samples within positive voters that are labeled positive at round *k* vote *j* are used to train the classifier $H_{k,j}$. Votes are then obtained for each sample *I* in each window within the candidate group, denoted $v_{i}^{k, j}\in[0, 1]$. Sample labels *Y* are then updated using the preferred voting selection method to be used in subsequent rounds of voting, unless a stopping criterion is met in which the algorithm will complete its process.

SUPPLEMENTARY FIGURE 12.


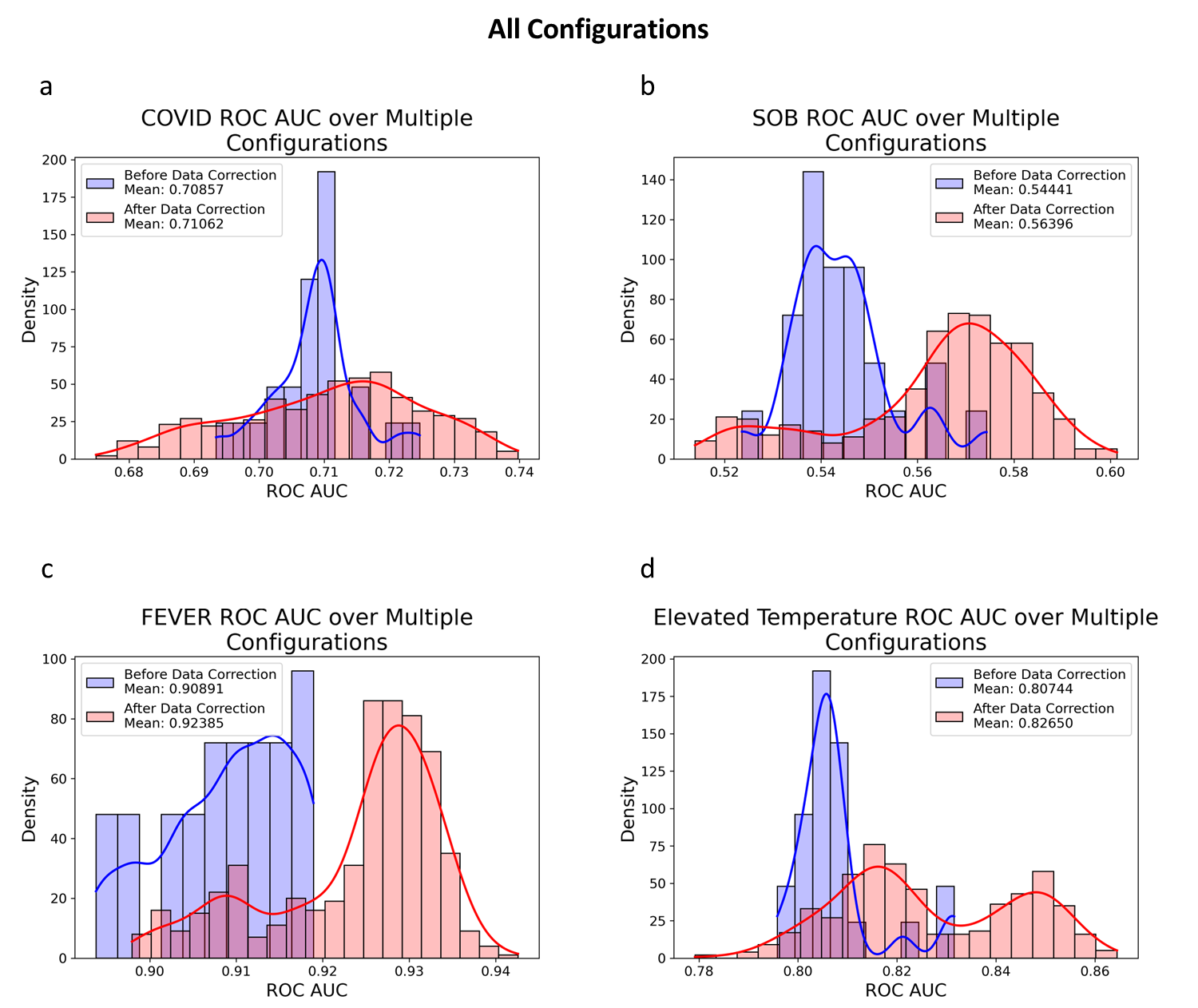


**Supplementary Figure 12.** **ROC AUC performance histograms across multiple XGBoost configurations.** The plots show the performance results for **a**, COVID, **b**, shortness of breath, **c**, fever, and **d**, elevated temperature. All configurations assessed are outlined in the Methods section under Performance Evaluation (n=576). A statistically significant shift can be seen when data correction methods are applied to the dataset prior to training and testing the machine learning model across all symptoms and COVID-19 detection. P-values of t-tests of two independent distributions for each histogram are as follows: **a**) p = 0.0019, **b**) p = 1.41e-83, **c**) p = 1.42e-142, **d**) p = 1.07e-94

SUPPLEMENTARY FIGURE 13.


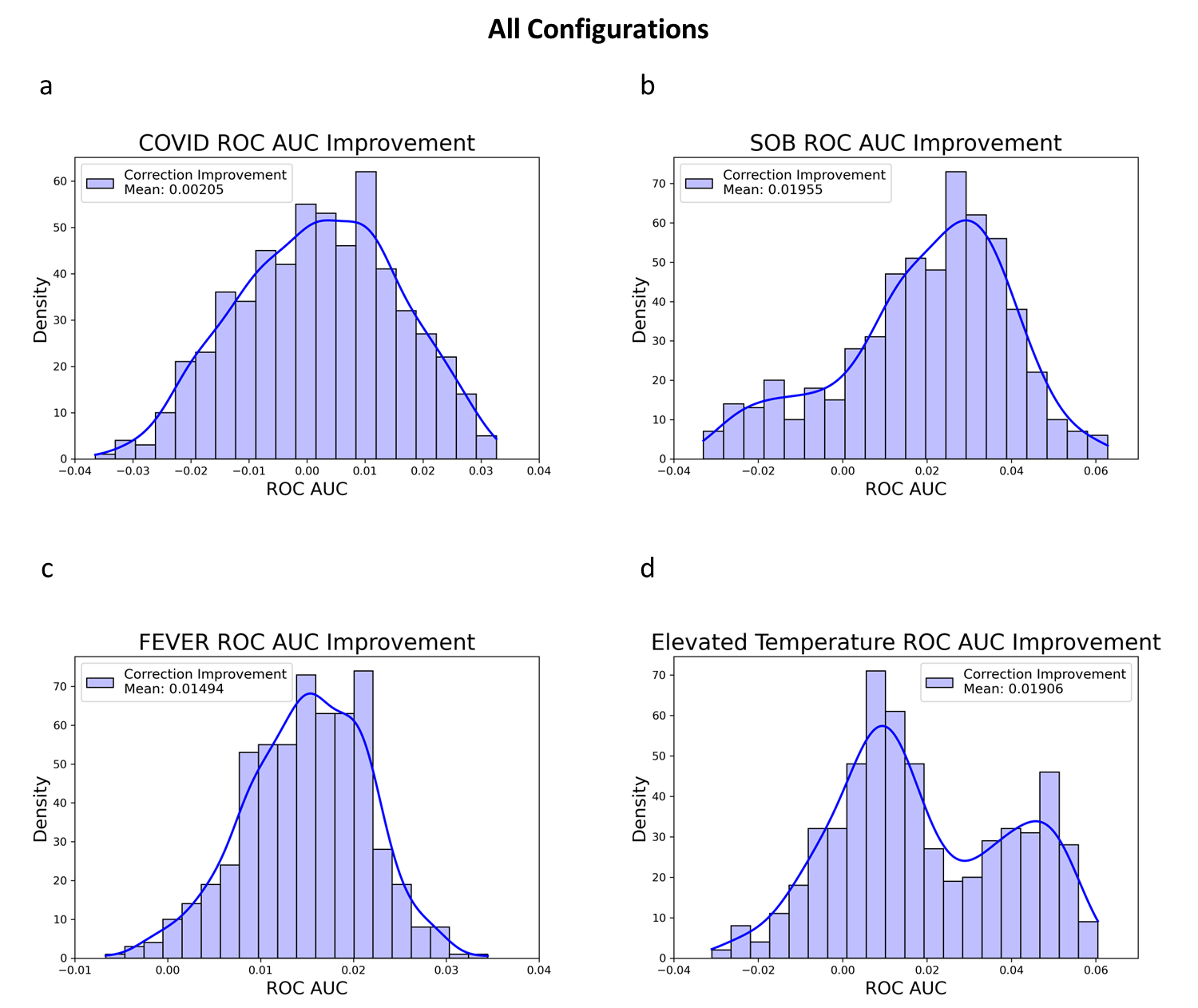


**Supplementary Figure 13.** **ROC AUC improvement histograms across multiple XGBoost configurations.** The plots show the performance results for **a**, COVID, **b**, shortness of breath, **c**, fever, and **d**, elevated temperature. All configurations assessed are outlined in the Methods section under Performance Evaluation (n=576). This is calculated by subtracting the ROC AUC after data correction from the ROC AUC before data correction for the same configurations. There is a statistically significant positive shift in improvement when data correction methods are applied to the dataset prior to machine learning training and testing. P-values of t-tests of one independent distributions with respect to an expected value (mean) of 0 for each histogram are as follows: **a**) p = 3.15e-4, **b**) p = 2.24e-86, **c**) p = 3.01e-229, **d**) p = 5.15e-79

SUPPLEMENTARY FIGURE 14.


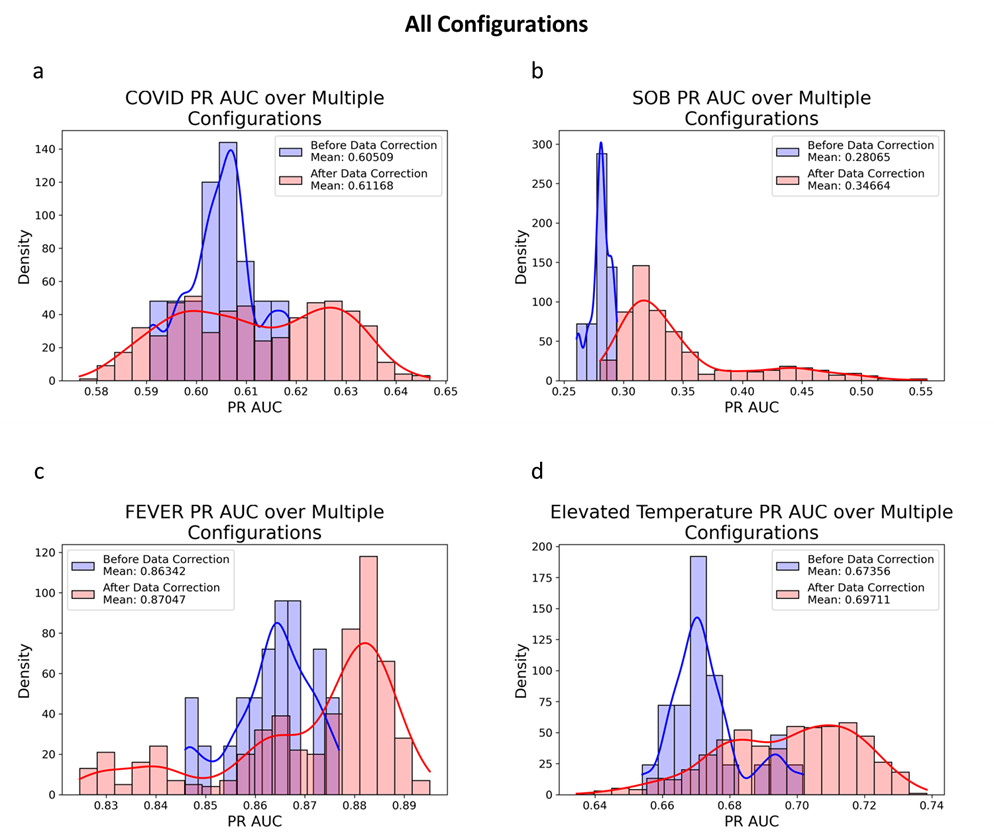


**Supplementary Figure 14.** **PR AUC performance histograms across multiple XGBoost configurations**. The plots show the performance results for **a**, COVID, **b**, shortness of breath, **c**, fever, and **d**, elevated temperature. All configurations assessed are outlined in the Methods section under Performance Evaluation (n=576). A statistically significant shift can be seen when data correction methods are applied to the dataset prior to training and testing the machine learning model across all symptoms and COVID-19 detection. P-values of t-tests of two independent distributions for each histogram are as follows: **a**) p = 1.13e-19, **b**) p = 9.67e-136, **c**) p = 1.07e-17, **d**) p = 1.46e-109

SUPPLEMENTARY FIGURE 15.


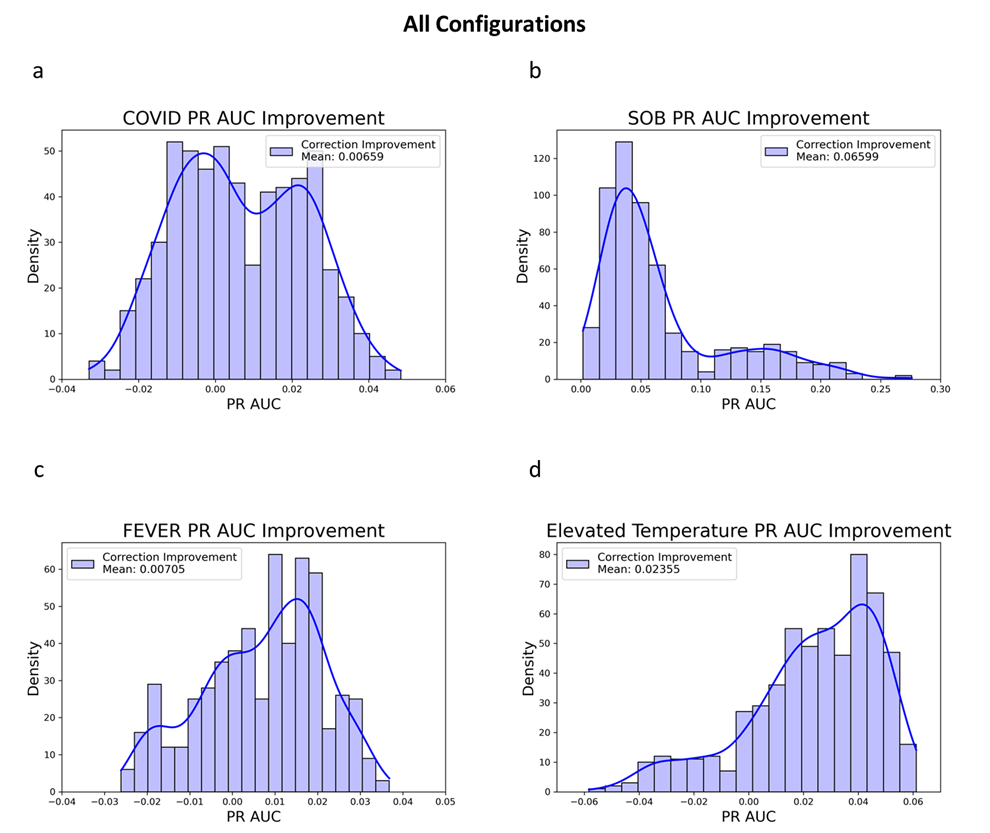


**Supplementary Figure 15.** **PR AUC improvement histograms across multiple XGBoost configurations.** The plots show the performance results for **a**, COVID, **b**, shortness of breath, **c**, fever, and **d**, elevated temperature. All configurations assessed are outlined in the Methods section under Performance Evaluation (n=576). This is calculated by subtracting the PR AUC after data correction from the PR AUC before data correction of the same configurations. There is a statistically significant positive shift in improvement when data correction methods are applied to the dataset prior to machine learning training and testing. P-values of t-tests of one independent distributions with respect to an expected value (mean) of 0 for each histogram are as follows: **a**) p = 9.11e-20, **b**) p = 1.09e-118, **c**) p = 7.46e-30, **d**) p = 3.07e-86

SUPPLEMENTARY FIGURE 16.


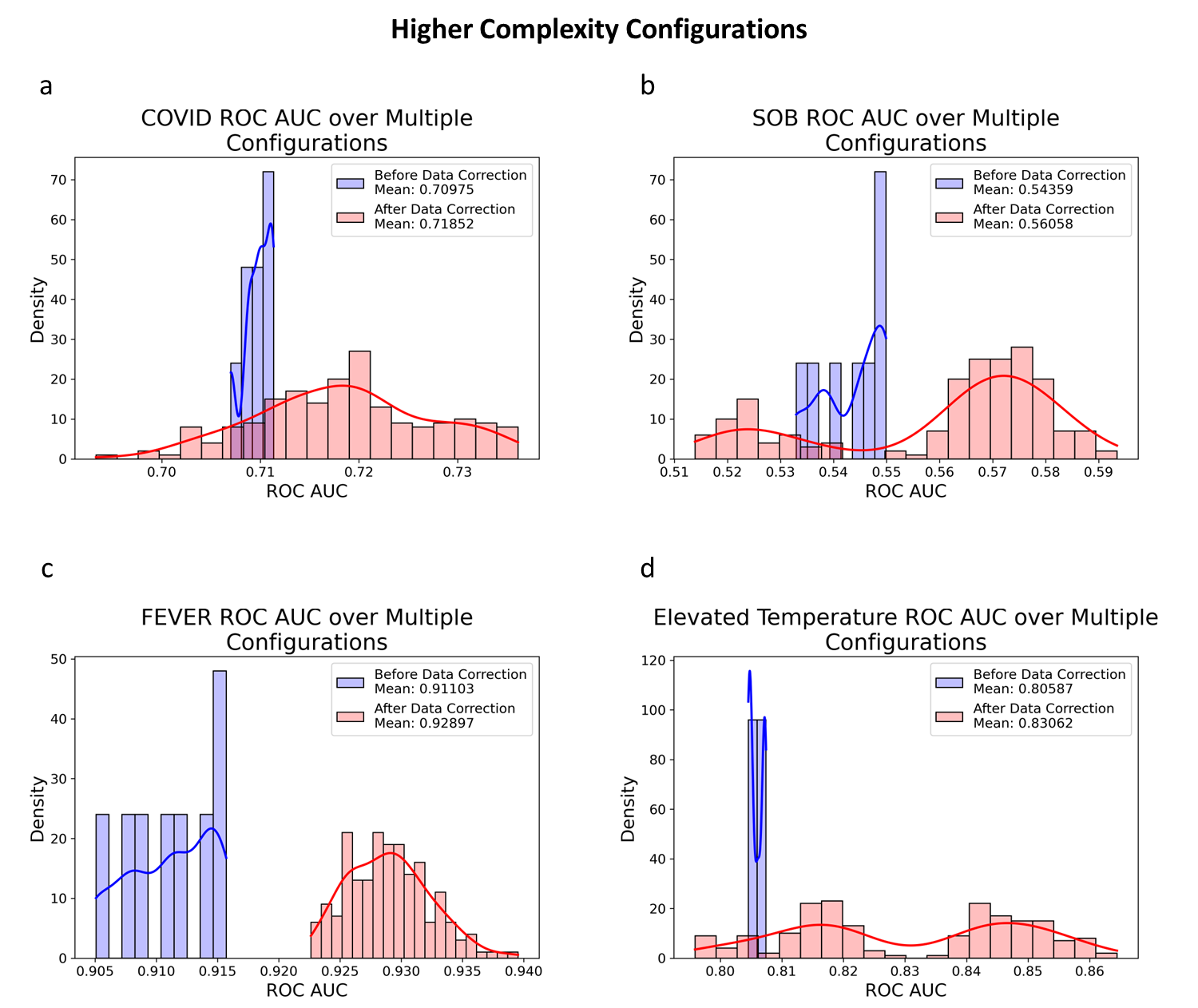


**Supplementary Figure 16.** **ROC AUC performance histograms across higher complexity XGBoost configurations**. The plots show the performance results for **a**, COVID, **b**, shortness of breath, **c**, fever, and **d**, elevated temperature. The configurations assessed are a subset of the total set of configurations, where the number of estimators range from 25 to 200 and the maximum tree depth ranges from 6 to 8 (n=192). This figure is similar to Supplementary Figure 12 with the exception of omitting the lower complexity XGBoost configurations to show that model performance significantly improves at higher complexity models. P-values of t-tests of two independent distributions for each histogram are as follows: **a**) p = 1.68e-34, **b**) p = 1.77e-22, **c**) p = 5.82e-173, **d**) p = 3.79e-54

SUPPLEMENTARY FIGURE 17.


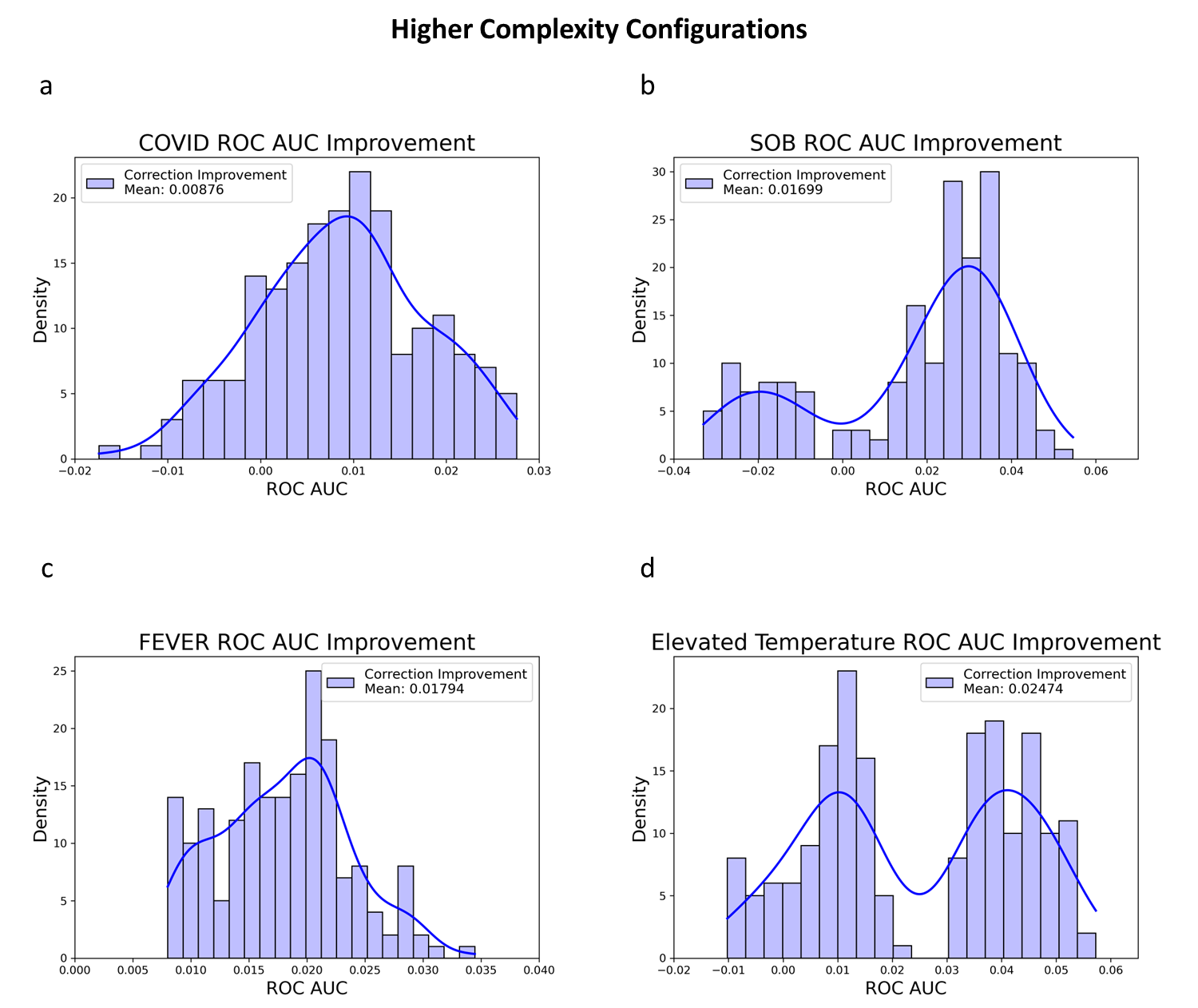


**Supplementary Figure 17.** **ROC AUC improvement histograms across multiple XGBoost configurations**. The plots show the performance results for **a**, COVID, **b**, shortness of breath, **c**, fever, and **d**, elevated temperature. The configurations assessed are a subset of the total set of configurations, where the number of estimators range from 25 to 200 and the maximum tree depth ranges from 6 to 8 (n=192). This is calculated by subtracting the ROC AUC after data correction from the ROC AUC before data correction of the same configurations. This figure is similar to Supplementary Figure 13 with the exception of omitting the lower complexity XGBoost configurations to show that model performance significantly improves at higher complexity models over lower complexities. P-values of t-tests of one independent distributions with respect to an expected value (mean) of 0 for each histogram are as follows: **a**) p = 9.95e-30, **b**) p = 2.02e-20, **c**) p = 2.65e-102, **d**) p = 5.78e-44

SUPPLEMENTARY FIGURE 18.


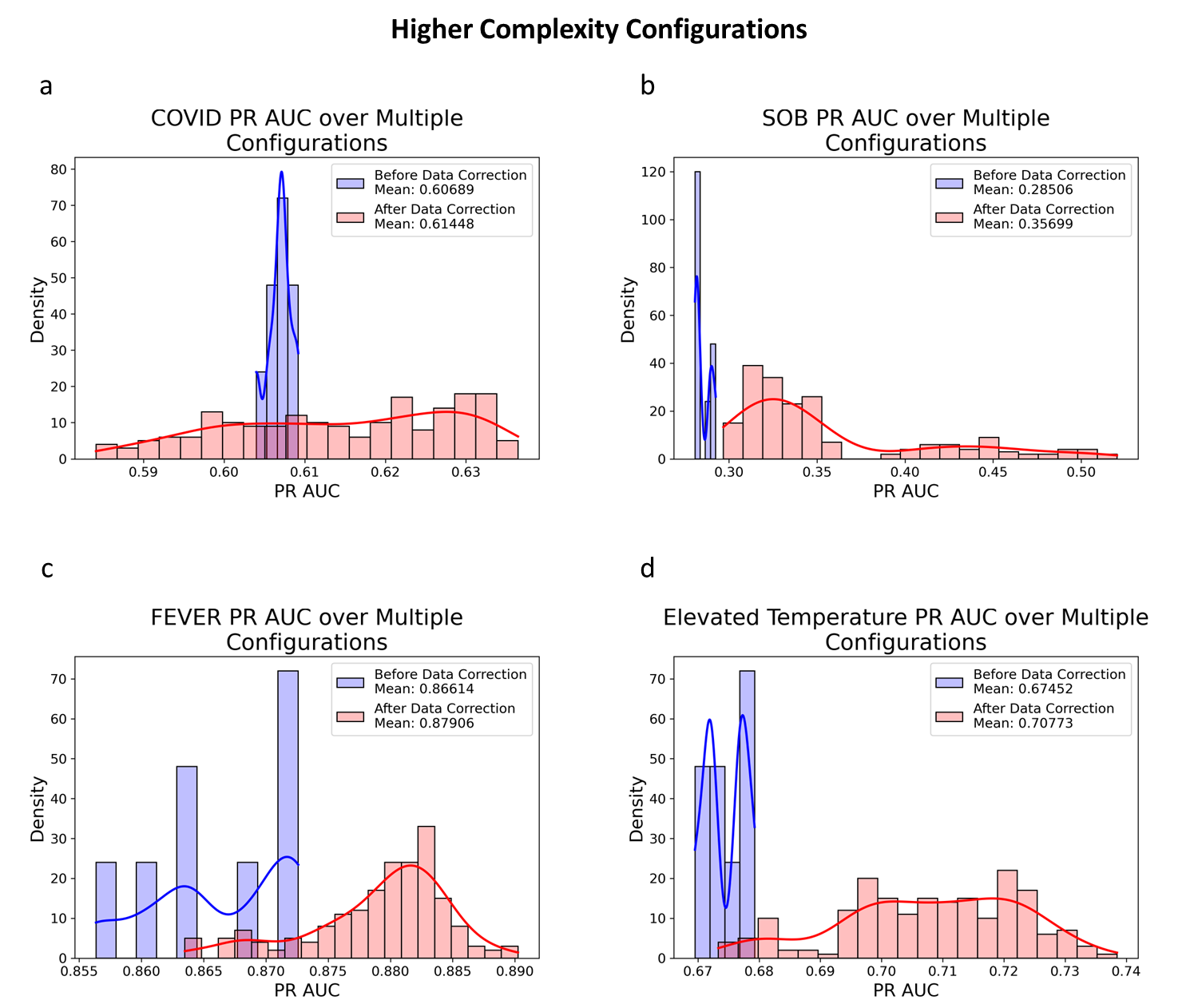


**Supplementary Figure 18.** **PR AUC performance histograms across higher complexity XGBoost configurations**. The plots show the performance results for **a**, COVID, **b**, shortness of breath, **c**, fever, and **d**, elevated temperature. The configurations assessed are a subset of the total set of configurations, where the number of estimators range from 25 to 200 and the maximum tree depth ranges from 6 to 8 (n=192). This figure is similar to Supplementary Figure 14 with the exception of omitting the lower complexity XGBoost configurations to show that model performance significantly improves at higher complexity models. P-values of t-tests of two independent distributions for each histogram are as follows: **a**) p = 5.49e-13, **b**) p = 2.93e-50, **c**) p = 1.64e-72, **d**) p = 2.10e-105

SUPPLEMENTARY FIGURE 19.


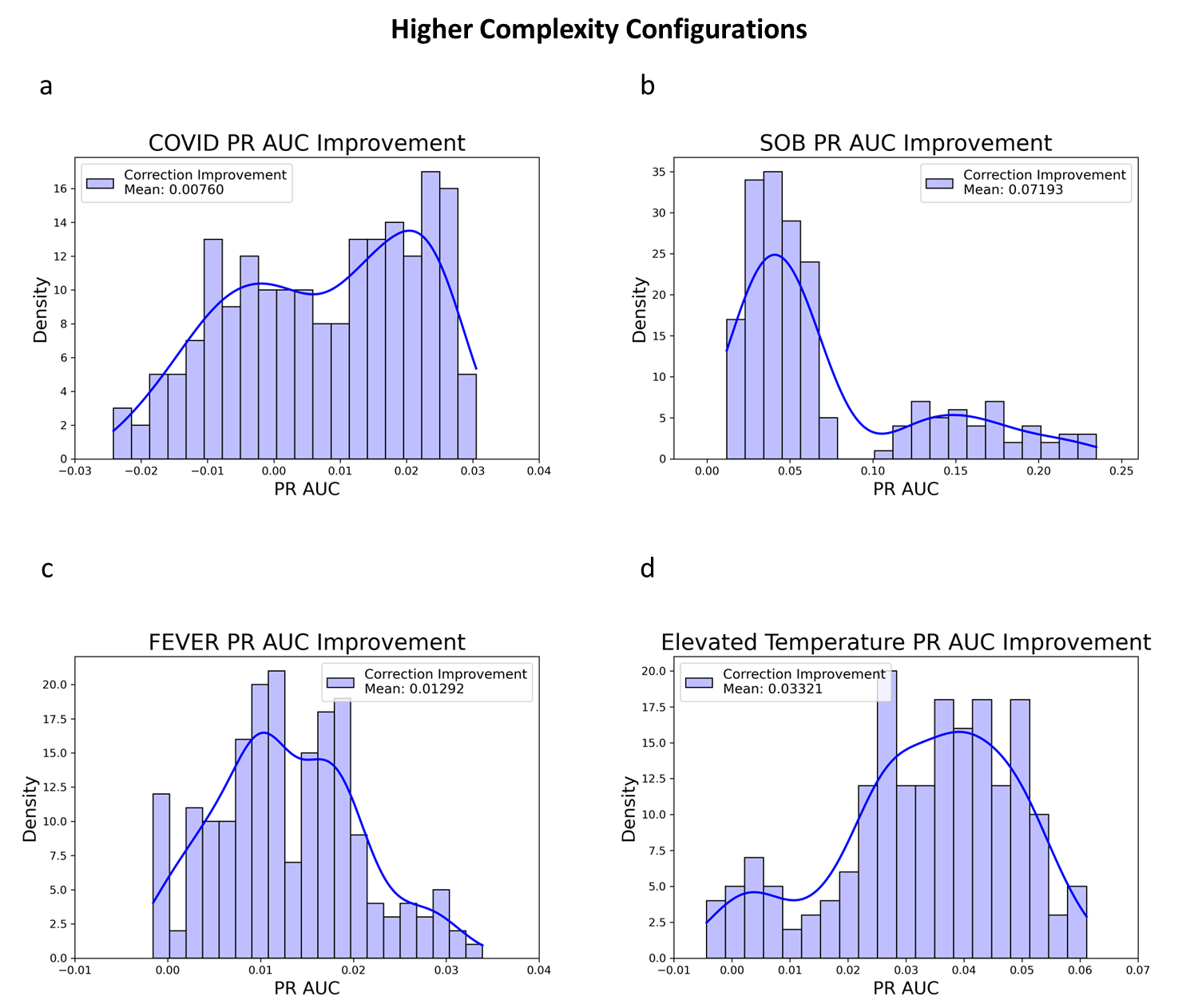


**Supplementary Figure 19.** **PR AUC improvement histograms across multiple XGBoost configurations.** The plots show the performance results for **a**, COVID, **b**, shortness of breath, **c**, fever, and **d**, elevated temperature. The configurations assessed are a subset of the total set of configurations, where the number of estimators range from 25 to 200 and the maximum tree depth ranges from 6 to 8 (n=192). This is calculated by subtracting the PR AUC after data correction from the PR AUC before data correction of the same configurations. This figure is similar to Supplementary Figure 15 with the exception of omitting the lower complexity XGBoost configurations to show that model performance significantly improves at higher complexity models over lower complexities. P-values of t-tests of one independent distributions with respect to an expected value (mean) of 0 for each histogram are as follows: **a**) p = 2.48e-12, **b**) p = 1.77e-41, **c**) p = 1.69e-57, **d**) p = 2.51e-74

SUPPLEMENTARY FIGURE 20.


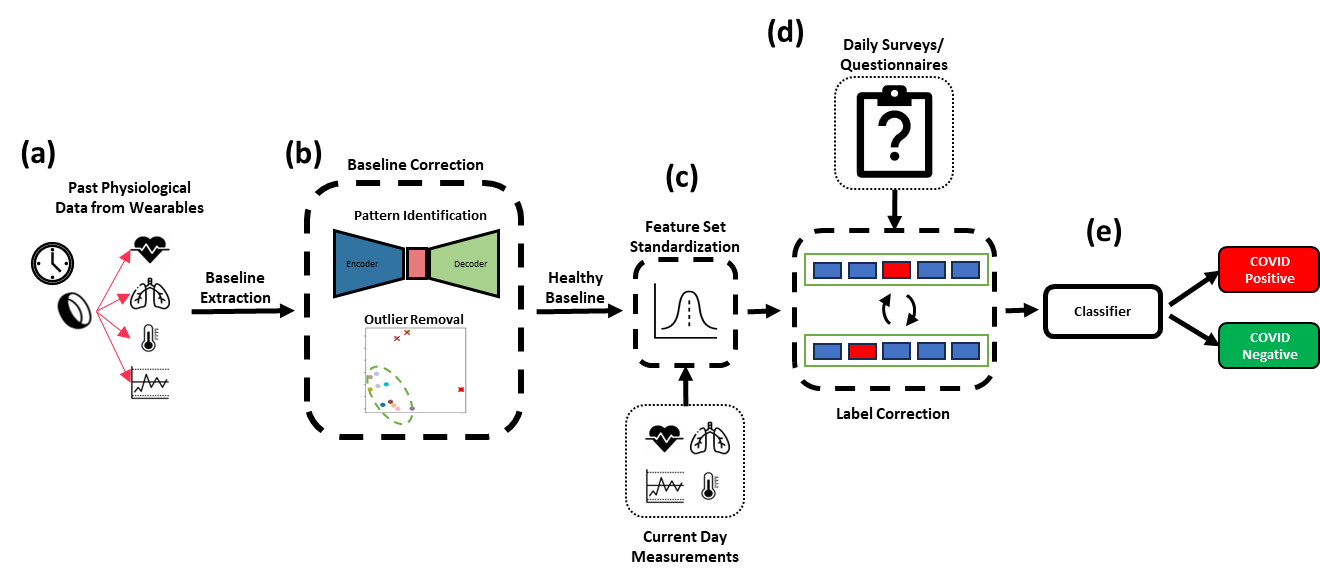


**Supplementary Figure 20.** **Overall methodology of infection classification.** **a**, physiological data is collected and extracted from COTS wearables and past data is extracted to form a baseline. **b**, baseline correction is performed for each baseline window, removing anomalous data. **c**, the healthy baselines are then used to normalize current day measurements (data taken the day of for infection prediction). **d**, during training of the ML algorithm, labels captured from daily surveys are corrected and used for training the XGBoost model to detect infections more accurately. **e**, the classifier, in this case XGBoost, predicts whether the physiological data is in response to an infection or not. More details on baseline correction and label correction can be found in Supplementary Figure 21, and Supplementary Figure 22, respectively.

SUPPLEMENTARY FIGURE 21.


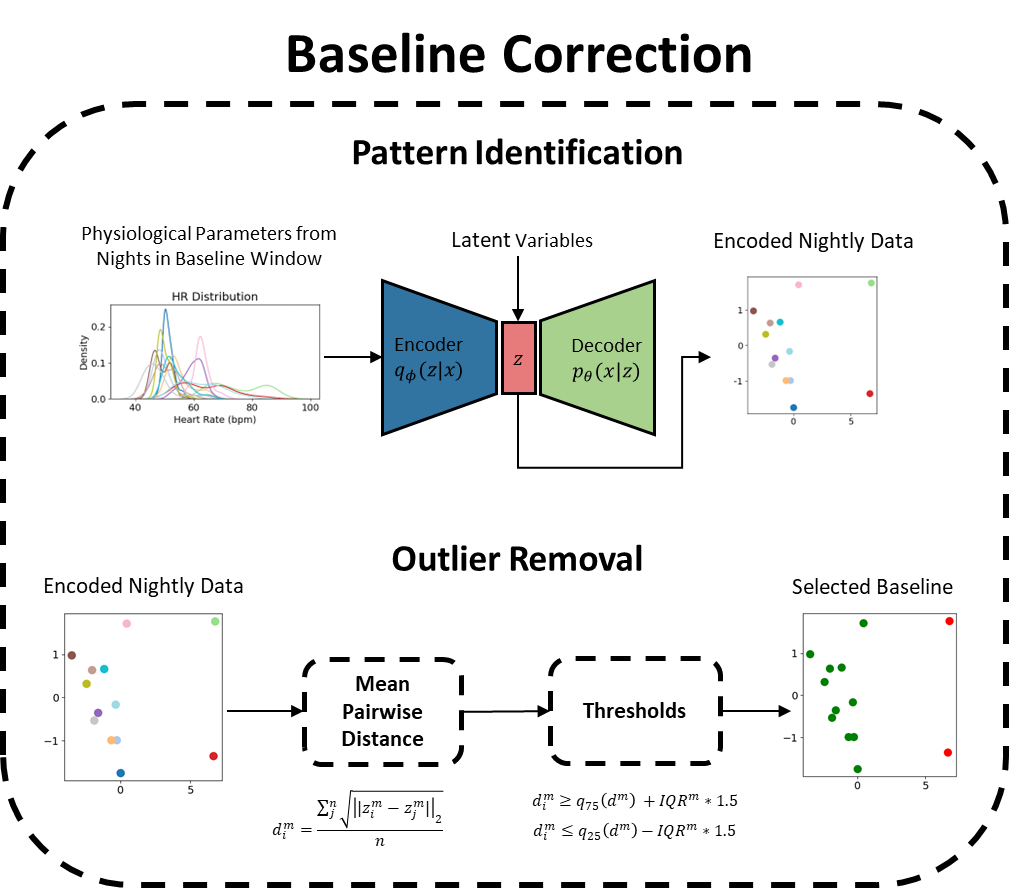


**Supplementary Figure 21. Baseline correction algorithm.** Baseline correction consists of two main components, pattern identification which utilizes a variational autoencoder (VAE) to compress the physiological raw data into latent variables, and outlier removal which takes the encoded latent variables and removes any outliers/anomalies present in a particular baseline. During pattern identification, physiological parameters from the nightly data of a participant’s baseline window are compressed into latent variables using a VAE to compress the most confounding data into fewer, more interpretable variables. This allows more direct comparison of night-to-night data and allows the use of trivial distance comparisons between nightly data. Outlier removal uses mean pairwise distance comparison combined with thresholding – in this case IQR outliers – to remove nights with more variational differences from the majority consensus. We assume that the majority consensus is that of a “healthy” baseline since a person is more likely to experience more normal physiological data over the course of a few weeks versus irregular responses. For illustrative purposes, we have shown heart rate feature in this figure, but the method is systematically applied to all features of consideration.

SUPPLEMENTARY FIGURE 22.

**
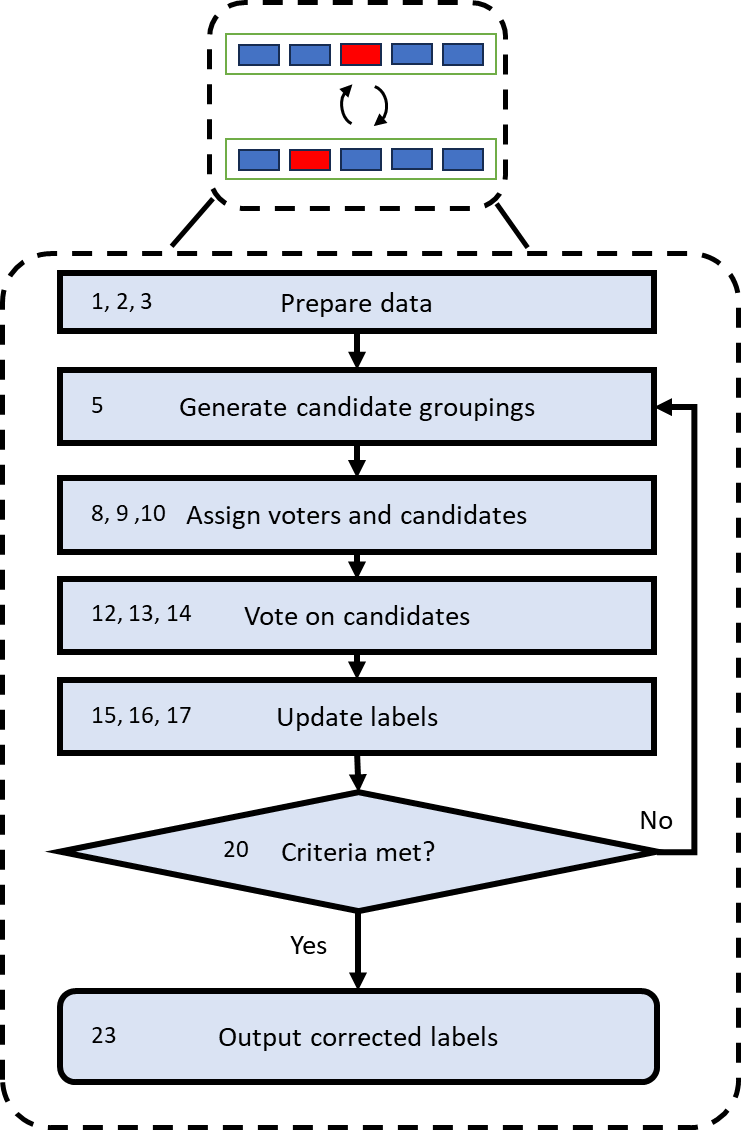
**

**Supplementary Figure 22. A flow diagram of the proposed probability voting algorithm.** The numbers in each box correspond to the lines shown in Algorithm 1. First, data is prepared through preprocessing and window generation (lines 1, 2, 3). The voting process then begins through the grouping of candidate windows (line 5), the training of a classifier (lines 8, 9, 10) to then vote on each candidate (lines 12, 13, 14), and then updating the labels in each candidate window (lines 15, 16, 17). If a stopping criterion is met (line 20) the algorithm outputs the corrected labels (line 23). If the stopping criterion is not met, the voting process continues until it is.

SUPPLEMENTARY REFERENCES

1. Puhach, O., Meyer, B. & Eckerle, I. SARS-CoV-2 viral load and shedding kinetics. Nat Rev Microbiol 21, 147–161 (2023). https://doi.org/10.1038/s41579-022-00822-w
2. Musa, S.S., Zhao, S., Wang, M.H. et al. Estimation of exponential growth rate and basic reproduction number of the coronavirus disease 2019 (COVID-19) in Africa. Infect Dis Poverty 9, 96 (2020). https://doi.org/10.1186/s40249-020-00718-y
3. Liang, Kaihao. “Mathematical model of infection kinetics and its analysis for COVID-19, SARS and MERS.” Infection, genetics and evolution : journal of molecular epidemiology and evolutionary genetics in infectious diseases vol. 82 (2020): 104306. doi:10.1016/j.meegid.2020.104306
